# Beyond classification metrics: a psychometric-aware benchmark for data augmentation in imbalanced student mental health surveys

**DOI:** 10.3389/fdgth.2026.1865324

**Published:** 2026-06-15

**Authors:** Chen Shao, Shengnan Qiao, Ang Li, Shipeng Liu, Yanglong Chen, Yuzhe Tan, Zhibo Liang, Xuanming Si

**Affiliations:** School of Intelligent Engineering, Shandong Management University, Jinan, China

**Keywords:** class imbalance, DASS-42, data augmentation, depression screening, GAN, imbalanced learning, machine learning, PHQ-9

## Abstract

**Background:**

Machine-learning-based depression screening from student survey data complements clinician assessment but faces two obstacles: class imbalance (causing under-prediction of urgent minority cases) and the untested assumption that synthetic augmentation data preserve psychometric validity. Although recent work has begun to evaluate distributional fidelity of synthetic survey data, no study has systematically benchmarked both classification utility and classical construct-validity measures on augmented student-mental-health datasets.

**Objective:**

Benchmark eight data-augmentation strategies on their classification utility and construct validity using a dual-axis evaluation framework.

**Methods:**

We evaluated no augmentation, random oversampling, SMOTE, BorderlineSMOTE, ADASYN, CTGAN, TVAE, and the proposed Psychometric-Constrained Tabular GAN (PCT-GAN) on three public datasets (PHQ-9: *n* = 682; Student Depression: *n* = 27,837; DASS-42: *n* = 26,459) using four classifiers (Random Forest, XGBoost, SVM, MLP) under 5-fold cross-validation. PCT-GAN extends conditional Wasserstein-GAN-with-gradient-penalty with two psychometric regularisers: (1) factor-reconstruction loss preserving item principal-component structure, and (2) correlation-preservation loss minimizing inter-item correlation deviation. Classification performance was assessed using Macro-F1, ROC-AUC, G-mean, and MCC; psychometric validity via Cronbach's *α*, Tucker's Congruence Coefficient, and Frobenius-norm deviation.

**Results:**

Overall, the eight methods were not statistically distinguishable on Macro-F1 (Friedman *χ*^2^ = 10.44, *p* = 0.17, *k* = 8, *N* = 12). However, dataset-specific patterns emerged: PCT-GAN + Random Forest achieved the largest gain on small, imbalanced PHQ-9 data (F1 = 0.814 vs. 0.785 NoAug); SMOTE variants dominated on large DASS-42 data with neural networks (F1 > 0.99). On psychometric validity, PCT-GAN reduced inter-item correlation deviation by 15% vs. TVAE and 31% vs. CTGAN, while maintaining direction-correct Cronbach's *α* (mean |Δ*α*| = 0.045). TVAE, by contrast, inflated *α* above the real-data ceiling on all three DASS-42 subscales—a marker of psychometric invalidity rather than superior fidelity. Pareto analysis identified PCT-GAN as the only method ranking in the top three for both classification utility and psychometric fidelity.

**Conclusions:**

Embedding construct-validity constraints into generative models produces synthetic mental-health data that is simultaneously useful for prediction and trustworthy for secondary research reuse. Practitioners should select SMOTE-family oversamplers for transient training-set augmentation, and PCT-GAN when synthetic data will be shared, re-analysed, or pooled with real samples for downstream psychometric research.

## 1 Introduction

### 1.1 Background and motivation

Mental-health deterioration among university students has become a worldwide public-health priority. Large-scale institutional data show consistently rising prevalence of depression, anxiety, and stress disorders on college campuses, with post-pandemic cohorts exhibiting symptom levels that exceed clinically actionable thresholds for a substantial minority of the student population (2, 3). Traditional clinician-administered assessment pipelines—face-to-face structured interviews, psychiatric referrals, follow-up visits—are difficult to scale at the speed with which student cohorts enter and leave the university every year. Against this back-drop, machine-learning (ML) based screening from routinely collected survey data has emerged as a pragmatic first-line tool: by predicting the likely severity category of an individual from a brief set of self-report items and contextual features, an ML screener can triage thousands of students in seconds and flag those who would most benefit from a clinician's attention (4, 5).

Contemporary ML screeners are typically trained on tabular datasets that combine standardised psychometric instruments—most commonly the nine-item Patient Health Questionnaire (PHQ-9) for depression and the 42-item Depression, Anxiety and Stress Scale (DASS-42)—with demographic and academic context variables. A growing body of benchmark studies has demonstrated the feasibility of such screeners on heterogeneous populations ranging from primary-care adults (4) to adolescents (3), and from classical depression targets (6) to emerging biomedical modalities such as virtual-reality-evoked physiological signals (5). As ML screening moves from the laboratory to real clinical settings, however, two methodological problems become increasingly visible: class imbalance on the data side, and construct validity on the measurement side. Resolving their interaction is the subject of the present study.

Mental-health survey datasets are, almost by definition, class-imbalanced. In the typical clinical distribution, a large majority of respondents report minimal or mild symptoms while the severe and moderately-severe categories—precisely those most clinically urgent—account for less than 20% of the sample. In our own benchmark datasets the imbalance ratio ranges from 1.41:1 (near-balanced binary task in D2) to 3.62:1 (five-class DASS-42 distribution in D3). We acknowledge that these ratios represent a moderate imbalance regime; in real-world psychiatric screening for acute conditions such as active suicidal ideation, minority classes can constitute fewer than 5% of the screened population, yielding ratios of 10:1 or greater (7, 8). Nevertheless, even moderate imbalance is sufficient to degrade minority-class recall substantially. Off-the-shelf classifiers trained on such data systematically under-predict the minority classes, producing screeners that miss the very students they are most designed to identify (7, 9). This failure mode is a general phenomenon in health-related classification: Pardamean et al. (10), for example, reported a 20-point drop in sensitivity for the rarest sleep stage when imbalance was left untreated, and similar losses are documented for cyberbullying detection (9), traumatic-brain-injury prognostication (7), and thalassemia-related mental-health classification (6).

The dominant family of remedies is data augmentation, an umbrella term that covers both statistical oversamplers such as SMOTE, BorderlineSMOTE and ADASYN and modern deep generative models such as CTGAN and TVAE. Both families have been shown to lift minority-class recall on depression-related tasks (11, 12), and comprehensive surveys continue to expand the methodological toolbox (13–15). Yet most of this prior work has been carried out in application domains—text, images, biomedical signals, industrial monitoring—whose data exhibit very different geometric and statistical regularities from the item-level psychometric data that dominate mental-health surveys. A systematic, psychometric-scale-aware benchmark of data-augmentation strategies for student-mental-health classification has therefore been conspicuously absent from the literature.

### 1.2 A hidden limitation: construct validity of synthetic data

A second, more subtle limitation of existing augmentation benchmarks is that they report only classification metrics (accuracy, F1, AUC). This unidimensional view is well suited to application areas where the synthetic data are transient training fuel and are never re-used downstream. But in psychological research the data themselves have a second, distinct life: item-level responses to validated scales are routinely reused for factor-structure analysis, reliability estimation, measurement-invariance testing, and meta-analysis. In such secondary analyses, construct validity—the degree to which the observed variables actually measure the latent construct they are intended to measure—is the gate-keeping property of data (16, 17). A synthetic sample that elevates Macro-F1 by two points but destroys the factor structure of the PHQ-9 or the inter-item correlation pattern of the DASS-42 produces data that cannot responsibly be shared, re-analysed or pooled with real samples.

Despite the obvious tension between classification utility and psychometric fidelity, systematic measurement of both axes on the same augmented student-mental-health datasets has remained largely absent. A partial exception is Jiang et al. (1), who proposed a framework for evaluating synthetic survey data and assessed distributional fidelity, but did not apply classical construct-validity measures such as Cronbach's *α*, Tucker's Congruence Coefficient, or inter-item correlation preservation. Augmentation surveys in adjacent fields (15, 18) discuss domain-specific fidelity criteria—temporal coherence in time-series, perceptual realism in images—but stop short of addressing the psychometric requirements of survey data. On the psychometrics side, the literature on construct validity (19–22) has developed sophisticated tools for assessing new scales, but these tools have rarely been applied to evaluate synthetic samples. The present study is, to the best of our knowledge, the first systematic attempt to benchmark classification utility and psychometric validity simultaneously on augmented student-mental-health survey data, and the research gap that motivates it lies precisely at this intersection.

### 1.3 Research questions, contributions and paper roadmap

To address the gap identified above, this work pursues three research questions:

RQ1—Classification utility. How do eight representative data-augmentation strategies (NoAug, Random Oversampling, SMOTE, BorderlineSMOTE, ADASYN, CTGAN, TVAE, and the proposed PCT-GAN) compare on Macro-F1, ROC-AUC, G-mean and Matthews' Correlation Coefficient across three student-mental-health datasets that differ in sample size, imbalance ratio and scale dimensionality?

RQ2—Psychometric validity. To what extent do deep generative augmentation methods preserve the three pillars of psychometric validity of the underlying scale, namely (i) internal consistency (Cronbach's *α*), (ii) factor-structure equivalence (Tucker's Congruence Coefficient), and (iii) inter-item correlation structure (Frobenius-norm deviation)?

RQ3—Performance-validity trade-off. Does a single augmentation strategy occupy the Pareto frontier of both evaluation axes simultaneously, or is high classification utility necessarily achieved at the cost of psychometric fidelity?

In answering these questions the paper makes four contributions:

C1—A systematic benchmark. We construct a factorially balanced benchmark of 8 augmentation methods × 4 classifiers × 3 datasets, evaluated under identical 5-fold cross-validation and a 20% held-out test set. To our knowledge, this is the most comprehensive head-to-head augmentation benchmark reported on student-mental-health data to date.

C2—The proposed PCT-GAN. We propose the Psychometric-Constrained Tabular GAN (PCT-GAN), a conditional WGAN-GP augmented with two novel regularisers: a factor-reconstruction loss that penalises deviations from the principal-component subspace of the real items, and a correlation-preservation loss that penalises the Frobenius distance between the generated and real inter-item correlation matrices. Unlike general-purpose tabular generators such as CTGAN and TVAE, whose training objectives carry no domain-specific inductive bias, PCT-GAN encodes construct-validity priors directly into the generator loss, bridging modern generative modelling and classical psychometric theory. The two regularisers are fully differentiable and integrate into standard generator training without additional hyper-parameter tuning.

C3—A dual evaluation framework. We formalise a dual evaluation protocol that combines four imbalance-aware classification metrics with three psychometric-validity indicators, and we demonstrate—through Friedman omnibus testing and Nemenyi critical-difference diagrams—that single-metric benchmarking can produce null results even when pronounced methodological differences exist along other meaningful axes.

C4—Full reproducibility. All preprocessing scripts, augmentation implementations, PCT-GAN training code, evaluation pipelines, random seeds and experimental results are released in a public repository, together with a reproducibility protocol that specifies every version number and hardware detail needed to re-execute the study end-to-end.

The empirical findings that follow from these contributions are striking. When classification utility is treated as the sole evaluation axis, eight augmentation strategies are statistically indistinguishable on our benchmark (Friedman *χ*^2^ = 10.44, *p* = 0.17). When psychometric validity is added as a second axis, however, the three generative methods diverge sharply: CTGAN collapses internal consistency on the Anxiety subscale (*α* = 0.626 vs. real: 0.910); TVAE inflates *α* above the real-data ceiling—a marker of psychometric invalidity, not superior fidelity; and PCT-GAN achieves a 31% improvement in correlation-structure preservation over CTGAN (paired bootstrap *p* < 0.0001) while keeping Cronbach's *α* direction-correct (mean |Δ*α*| = 0.045). A Pareto analysis across the two axes identifies PCT-GAN as the only method that simultaneously ranks in the top three on classification and the top one on psychometric fidelity—an outcome that directly supports the central methodological argument of this study: evaluating synthetic psychometric data by classification metrics alone is incomplete, and constraint-aware generators are needed when the synthetic samples will be reused for secondary psychological research.

The remainder of the paper is organised as follows. Section 2 reviews related work in ML-based mental-health screening, data-augmentation methodology for tabular data, and the psychometric-validity literature, and identifies the research gap in greater detail. Section 3 presents the three benchmark datasets and the complete methodology, including the proposed PCT-GAN model and the dual evaluation framework. Section 4 reports the empirical results. Section 5 discusses the implications, theoretical contributions and limitations of the study. Section 6 concludes.

## 2 Related work

The present study sits at the intersection of three research streams: (i) machine-learning-based mental-health classification, (ii) data-augmentation methods for imbalanced tabular data, and (iii) the psychometric-validity evaluation of survey instruments. Each stream has been active for decades in isolation, yet their convergence around synthetic psychometric data is very recent. This section reviews each stream in turn (Sections 2.1–2.3) and closes with an explicit research-gap synthesis (Section 2.4) that motivates the contributions announced in Section 1.

Review methodology. The literature search was conducted across five databases: PubMed, Web of Science, Scopus, IEEE Xplore and Google Scholar. Search terms comprised combinations of “data augmentation”, “class imbalance”, “mental health classification”, “depression screening”, “SMOTE”, “GAN”, “tabular synthetic data”, “psychometric validity”, “Cronbach's alpha” and “construct validity”. The primary date range was January 2019–March 2026, with seminal earlier works (e.g., foundational SMOTE and DASS-42 publications) included regardless of date. Inclusion criteria required that a work be (i) a peer-reviewed empirical or methodological paper and (ii) directly relevant to at least one of the three review streams above. Exclusion criteria removed conference abstracts without full papers, non-English publications, and papers focused exclusively on image or audio modalities with no tabular component.

### 2.1 Machine learning for mental-health classification

ML-based detection of depression and related mood disorders has matured into a broad, multi-modal research agenda. A recent systematic review of depression-related learning methods (23) identifies four dominant modalities: EEG, functional neuroimaging, physical and behavioural signals, and self-report survey data. Each modality has given rise to its own sub-literature, and each exhibits the twin challenges of small sample size and class imbalance that motivate the present benchmark.

On the neuro-signal side, EEG-based models have been proposed by Wei et al. (24), who combined trial-level synthesis with convolutional classifiers to improve depression recognition under limited-sample conditions, and fMRI-based spatio-temporal aggregation networks have been used by Zhang et al. (25) to explicitly target the “small-and-unbalanced” regime. Cross-modal generative models for fNIRS signals have also appeared (26), and larger multi-modal depression-detection pipelines are now emerging, exemplified by the Mamba-based architecture of Liu et al. (27) that fuses audio, facial-video and physiological streams.

On the behavioural and environmental side, skeleton-based gait information has been shown to be predictive of depressive symptomatology (28), and wearable/IoT systems have extended monitoring to naturalistic settings (29). Activity-based explainable models for severity classification have been developed by Ahmed et al. (30), while clinically grounded depression-prediction pipelines have been reported for community cohorts (31) and for hepatitis-B patients (32).

On the text side, transformer-based detectors applied to social-media posts have become the de-facto standard (33–35), and the integration of generative AI with psychological features for early social-media screening has been explored by Hsu et al. (36). For structured clinical data, Oh et al. (37) have recently released a DSM-5-aligned depression dataset that raises the standards for how clinical targets should be constructed in future benchmarks.

Two threads of this large literature are directly relevant for the present study. First, the interpretability thread (6, 38, 39) has made clear that mental-health screeners intended for clinical or educational deployment must yield classifiers that practitioners can understand and audit; this shapes our decision in Section 3.4 to compare heterogeneous, individually interpretable learners (RF, XGB, SVM, MLP) rather than opaque ensembles. Second, the imbalanced-data thread (25, 40) demonstrates that even state-of-the-art deep models falter when the minority class is severely under-represented, which in turn drives the dense recent activity on data augmentation reviewed in Section 2.2.

For student populations specifically, the literature is sparser but growing. Ma and Li (41) combined SMOTE with CNN-based painting-image clustering for art-student mental-health screening, and Dong and Huang (2) built a behavioural-feature model for Chinese college students using educational big-data mining. More generally, ML-based depression screeners trained on PHQ-style questionnaires have been reported across adult primary-care (4) and adolescent (3) cohorts. However, none of these studies has systematically compared modern data-augmentation strategies on multiple student-mental-health datasets while also quantifying the psychometric integrity of the generated samples—the specific gap the present benchmark is designed to close.

### 2.2 Data-augmentation methods for imbalanced tabular data

#### 2.2.1 Statistical oversampling

Synthetic Minority Oversampling (SMOTE) and its variants remain the workhorses of imbalanced-data classification. SMOTE has been applied to mental-health targets across an impressive breadth of settings: geriatric depression prediction from survey data (42), suicide-risk prediction from adult ADHD and depression indicators (8), handwriting-based schizophrenia and bipolar-disorder detection (43), and psychiatric clinical-notes classification (44). The BorderlineSMOTE variant concentrates synthetic points near the decision boundary and has been shown to outperform vanilla SMOTE on multimodal sleep-staging data (45). ADASYN, which adaptively generates more synthetic samples in regions of higher classification difficulty, has been adopted for multi-level depression classification (46) and for an Indonesian consumer-health-question classification task (47). Hybrid combinations of SMOTE with edited-nearest-neighbour cleaning or LASSO-based feature selection have further pushed the performance envelope (48, 78). Despite their popularity, however, none of these studies has asked whether the synthetic points generated by linear interpolation preserve the measurement-level properties of the underlying instrument—a question we return to in Section 2.3.

#### 2.2.2 Deep generative augmentation

Deep generative models have recently been promoted as a more expressive alternative to SMOTE for tabular data. Conditional Tabular GANs (CTGAN) have been applied to metabolomics-based CVD–depression co-morbidity diagnosis (49) and to obesity prediction from social physical-activity data (50). Tabular VAEs (TVAE) have been benchmarked for landslide-susceptibility prediction (51), and rigorous head-to-head comparisons of CTGAN and TVAE on synthetic-survey generation have been reported by Yadav et al. (52) and Jiang et al. (1). Generative models have also gained traction within the mental-health domain itself: Bang et al. (53) introduced DepressGEN, a GAN-style framework dedicated to depression-detection data generation, and generative-AI frameworks for assessment and treatment have been reviewed in adjacent fields such as autism spectrum disorder (54). A broader methodological survey of GANs in bio-electric medicine (55) and comprehensive augmentation surveys by Mumuni and Mumuni (13), Khalifa et al. (56), Bansal et al. (14), Jiang et al. (18) and Wang et al. (15) collectively document an acceleration of interest in deep tabular generators. Yet across all of these works, the evaluation protocol is invariably classification-performance-centric: a synthetic dataset is deemed “good” if it boosts downstream F1, and no further examination of its measurement-level validity is carried out.

#### 2.2.3 Text-based and modern augmentation

Complementary to statistical and deep-generative augmentation, text-based and LLM-driven augmentation has begun to appear in mental-health pipelines. Nilsson and Kovács (35) examined summarisation-based augmentation for social-media depression detection, Das et al. (57) proposed an iterative-training pipeline with text-based augmentation for mental-health classification, and Chai et al. (58) provide a comprehensive survey of text data augmentation in the era of large language models. For biomedical-signal modalities, Wei et al. (24) used synthesis-based augmentation for EEG-based depression detection, Yang et al. (28) applied domain-specific augmentation to skeleton-based gait features, and Shao et al. (26) designed a cross-modal augmentation scheme for fNIRS. Similar cross-domain transfers of augmentation ideas have been surveyed by Weng et al. (59) and Hong et al. (60). These modality-specific contributions reinforce the general observation that augmentation strategies that succeed in one domain do not automatically transfer to another; the tabular psychometric regime—dominated by ordinal Likert-scale items with strong factor structure—has its own peculiarities that demand method-specific evaluation.

### 2.3 Psychometric validity of survey data

Psychometric validity is the meta-property that determines whether an instrument actually measures the latent construct it purports to measure (16). Three overlapping facets of validity are operationalised in the classical literature: structural (factor-analytic evidence that the items cluster into the theorised factors), internal-consistency (Cronbach's *α* and related reliability coefficients), and convergent-divergent (correlations with external criteria). Applied-psychometric studies across the health sciences routinely triangulate these facets when validating new or translated instruments: cross-cultural validations of the SarQoL quality-of-life scale (20), functional-inability scales for dancers (61) and athletes (62), nursing-competence tools (19), decision-making questionnaires for physicians (63), autism-specific participation questionnaires (64), and outreach-dental-camp perception scales (65) all follow a remarkably consistent validation template: (i) CFA or EFA on a development sample, (ii) Cronbach's *α* per subscale, (iii) convergent-divergent tests with external constructs. The same template has been used to validate depression- and emotion-adjacent scales (17, 22) as well as large population-level instruments such as SF-12v2 (66) and the Oral-Frailty-Index-8 (21).

Across this entire body of psychometric work, the data on which validity is assessed are, without exception, real response data. The question of whether synthetic survey data generated by ML methods preserve these same validity properties has only very recently begun to receive attention. Jiang et al. (1) have proposed a general framework for generating and evaluating synthetic survey data, representing an important step toward bridging generative modelling and survey methodology; however, their evaluation focuses on distributional fidelity (e.g., marginal and joint distributions) rather than the classical psychometric facets of construct validity listed above. Similarly, Yadav et al. (52) provide distributional-fidelity comparisons of TVAE and CTGAN but do not assess internal consistency, factor-structure equivalence, or inter-item correlation preservation. This disconnect between modern generative modelling and classical psychometrics—specifically, the absence of Cronbach's *α*, Tucker's Congruence Coefficient, and Frobenius-norm deviation as evaluation criteria for synthetic survey data—is the precise gap that the dual evaluation framework of Section 3.5 is designed to fill.

### 2.4 Research-gap synthesis

Table 1 in the Appendix maps the 80 works reviewed in Sections 2.1–2.3 against three evaluation dimensions: classification utility, psychometric-validity and mental-health specificity. The resulting matrix yields three headline observations. First, mental-health ML studies (Section 2.1) routinely report classification metrics but almost never report psychometric-validity indicators on augmented or synthetic data. Second, data-augmentation methodology papers (Section 2.2) almost exclusively frame their improvements in classification-utility terms, among the works reviewed, none measures Cronbach's *α*, TCC, or inter-item correlation preservation on the generated samples, although Jiang et al. (1) have begun to address distributional fidelity in a related context Third, psychometric-validity papers (Section 2.3) operate entirely on real data, have well-developed evaluation metrics, but have yet to encounter synthetic data at scale. The intersection of the three sub-literatures—psychometric-validity-aware augmentation of tabular mental-health data—is the empty cell in the matrix, and it is the niche that this study seeks to occupy with (i) the proposed PCT-GAN, whose loss function explicitly encodes two of the three classical psychometric facets (factor structure and inter-item correlation), and (ii) the dual evaluation framework of Section 3.5, which benchmarks classification utility and psychometric validity on the same augmented data.

**Table 1:** Research-gap matrix: 80 works mapped against three evaluation dimensions.

Research stream	Section	N works	Reports classification utility?	Reports psychometric validity?	Mental-health specificity	Representative examples
ML in mental health	2.1	20	✓ All	✗ None	✓ High	Wei et al. (24), Liu et al. (27), Kerasiotis et al. (33), Cho et al. (31), Dong & Huang (2)
Data-augmentation methods	2.2	9	✓ All	✗ None	∼ Mixed	SMOTE-family papers (8, 42), CTGAN (49), TVAE (51)
Psychometric validity	2.3	6	✗ None	✓ All	∼ Some	Lee et al. (20), Osborn et al. (19), Correa et al. (21), SF-12v2 validation (66)
Cross-section reviews	2.1–2.3	45+ (reused)	✓ Selective	✗ Selective	✓ Mixed	Mumuni & Mumuni (13), Bansal et al. (14), Jiang, Zheng et al. (18), Wang, Wang et al. (15)

## 3 Methodology

This section presents the end-to-end methodology of the proposed benchmark. We first outline the overall experimental framework (Section 3.1), describe the three student mental-health datasets and the preprocessing pipeline (Section 3.2), and then formalise the eight data-augmentation strategies compared in this study, including the proposed Psychometric-Constrained Tabular Generative Adversarial Network (PCT-GAN) (Section 3.3). The four downstream classifiers (Section 3.4) and the dual evaluation framework combining classification metrics and psychometric-validity indicators (Section 3.5) are then detailed. Finally, the experimental protocol, statistical tests and reproducibility settings are specified in Section 3.6.

### 3.1 Overall framework

The study adopts a strictly block-structured experimental framework in which each augmentation method is embedded in an otherwise identical training–evaluation pipeline. For every dataset, the raw tabular records are first cleaned, encoded and split into a stratified training/validation/test partition. Within each cross-validation fold, one of eight augmentation strategies is applied only to the training fold; the validation and test folds are kept untouched to ensure that no synthetic sample leaks into performance estimation. The four classifiers are then trained on the resulting (possibly augmented) training set and evaluated on the held-out partitions. In parallel, for the three generative models (CTGAN, TVAE, PCT-GAN) the synthetic samples are passed through a psychometric-validity module that computes factor-loading agreement and inter-item correlation preservation with respect to the original training data. This dual track—classification utility on the one hand and construct-validity preservation on the other—constitutes the core novelty of the benchmark, and follows the framework for synthetic-survey evaluation recently advocated by Jiang et al. (1).

### 3.2 Datasets

Three publicly available datasets with markedly different sizes, dimensionalities and imbalance profiles are used. Table 2 summarises their key characteristics.

**Table 2:** Summary of the three benchmark datasets.

Characteristic	D1—PHQ-9 Enhanced	D2—Student Depression	D3—DASS-42 (student subset)
Source	Kaggle (5th Edition)	Kaggle (Indian universities)	Open Psychometrics
Psychometric scale	PHQ-9 (9 items)	— (no standardised scale)	DASS-42 (42 items)
Underlying factor structure	Unidimensional	—	Three factors (D, A, S)
Samples after preprocessing	682	27,837	26,459
Features (after leakage removal)	14	12	47
Label	PHQ-9 severity (5 classes)	Depression (binary)	DASS-42 depression severity (5 classes)
Class distribution	206/155/128/125/68	11,547/16,290	5,521/2,515/4,841/4,486/9,096
Imbalance ratio (max/min)	3.03	1.41	3.62
Age range	17–26	18–59 (students)	14–30 (filtered)
Leakage columns removed	PHQ_Total	—	depression_score, anxiety_score, stress_score

#### 3.2.1 D1—PHQ-9 enhanced dataset

The first dataset comprises 682 university-aged respondents (17–26 years) who completed the nine-item Patient Health Questionnaire (PHQ-9; 79) together with three contextual ordinal items (sleep quality, study pressure, financial pressure). The dataset is publicly available on Kaggle (PHQ-9 5th Edition, https://data.mendeley.com/datasets/kkzjk253cy). Each PHQ-9 item is recorded on the standard four-point response scale (“Not at all”, “Several days”, “More than half the days”, “Nearly every day”). The clinical label is a five-level severity rating (Minimal/Mild/Moderate/Moderately severe/Severe). The class distribution is imbalanced (maximum-to-minimum ratio ≈ 3.03), and the smallest class (Severe) contains only 68 samples, which makes D1 a challenging low-resource multi-class scenario. The PHQ-9 is one of the most widely validated depression-screening instruments in both primary-care and student populations (4).

#### 3.2.2 D2—student depression dataset

The second dataset contains 27 901 records of self-reported depression status collected across several Indian university cities (Kaggle Student Depression Dataset, https://www.kaggle.com/datasets/hopesb/student-depression-dataset). After restricting the cohort to respondents whose declared profession is “Student” (27,870 rows) and removing rows with missing core variables, 27 837 records remain. Each record includes twelve features spanning demographics (gender, age), academic indicators (CGPA, academic pressure, degree level, study satisfaction, work/study hours), lifestyle factors (sleep duration, dietary habits), mental-health history (suicidal ideation, family history) and economic stress (financial stress). The binary label “Depression” (0 = no, 1 = yes) is only mildly imbalanced (41.5%:58.5%), yet the large sample size enables a rigorous evaluation of augmentation utility under near-balanced conditions—an under-studied regime in prior depression-classification work (8). It should be noted that this binary outcome is derived from a single self-reported item rather than from a validated psychometric scale; consequently, the psychometric-validity analysis in Section 4.4 cannot be applied to D2, and this limitation is discussed further in Section 5.4.

#### 3.2.3 D3—DASS-42 student subset

The third dataset is derived from the Open Psychometrics DASS-42 corpus (*n* ≈ 39 775; https://www.kaggle.com/datasets/yashpra1010/dass-19). The 42-item Depression, Anxiety and Stress Scale (DASS-42) captures three theoretically distinct but empirically correlated constructs, making it an ideal testbed for multi-factor psychometric-validity analysis. Because the corpus mixes respondents of all ages and education levels, we restrict the sample to respondents with education level 2 (high school) or 3 (university degree) and age 14–30, yielding 26 459 students. Each original item is re-coded from the published 1–4 scale to the canonical DASS 0–3 scale (by subtracting 1) before the three 14-item subscale scores are summed. Following the official DASS-42 cut-offs (0–9 Normal; 10–13 Mild; 14–20 Moderate; 21–27 Severe; ≥28 Extremely Severe), a five-level depression severity label is produced. The complete class distribution after preprocessing is: Normal 5,521 (20.9%), Mild 2,515 (9.5%), Moderate 4,841 (18.3%), Severe 4,486 (17.0%), Extremely Severe 9,096 (34.4%). The smallest class is Mild (*n* = 2,515), yielding an imbalance ratio of max/min = 9,096/2,515 = 3.62. The pronounced over-representation of the Extremely Severe class reflects the self-selection bias typical of online psychometric surveys, which we revisit in the Discussion (Section 5.4).

#### 3.2.4 Preprocessing

All three datasets share the following preprocessing steps: (i) categorical strings are ordinal-encoded to preserve psychometric ordering (e.g., the four PHQ-9 response levels become {0, 1, 2, 3}); (ii) binary fields are mapped to {0, 1}; (iii) records with missing values in any core column are removed (dropna); (iv) total-score columns that are deterministic linear combinations of the individual items (PHQ_Total in D1; depression_score, anxiety_score, stress_score in D3) are explicitly excluded from the feature matrix to prevent target leakage; (v) all features are passed to downstream models in their native scale, with standardisation applied inside each classifier pipeline when required (Section 3.4). Class distributions, feature counts and imbalance ratios after preprocessing are listed in Table 2.

### 3.3 Data augmentation methods

Eight strategies spanning four methodological families are benchmarked. The choice reflects both the dominant baselines in imbalanced mental-health classification (12, 46) and the most recent synthetic-tabular-data generators (1, 52). Unless stated otherwise, every method is applied independently to the training fold of each cross-validation split, with the target sample size set to match the count of the majority class (full oversampling).

#### 3.3.1 No augmentation (NoAug, baseline)

The classifier is trained directly on the original imbalanced training fold. This baseline quantifies the intrinsic imbalance-induced performance gap and serves as a common reference for all subsequent comparisons.

#### 3.3.2 Random oversampling (ROS)

The minority-class samples are replicated uniformly at random until every class reaches the majority-class size. ROS introduces no novel information and is known to amplify over-fitting on the duplicated points, but remains a strong naive baseline (12).

#### 3.3.3 SMOTE

The Synthetic Minority Oversampling Technique (SMOTE) generates synthetic minority examples by linearly interpolating between a minority sample x and one of its k nearest minority neighbours in feature space. The classic formulation is

$$x_{\mathrm{new}}=x_{i}+\lambda (x_{j}-x_{i}),\;\lambda ∼U(0,1),$$

where $x_{j}$ is a neighbour of $x_{i}$ and λ is sampled uniformly in the unit interval. SMOTE is the single most widely adopted oversampling technique in mental-health classification (41, 67) and provides a strong, interpretable baseline against which generative methods can be evaluated. We set k=5, clipped to min(k,|minority|−1) when the minority count is small.

#### 3.3.4 BorderlineSMOTE

BorderlineSMOTE restricts interpolation to minority samples that lie close to the decision boundary, thereby concentrating synthetic points in regions where additional coverage is most informative for the classifier (45, 47). We adopt the “borderline-1” variant with k=5 and m=10.

#### 3.3.5 ADASYN

Adaptive Synthetic sampling (ADASYN) extends SMOTE by placing more synthetic samples in neighbourhoods where minority instances are surrounded by majority neighbours—i.e., locations of higher classification difficulty. ADASYN has been shown to outperform vanilla SMOTE in multi-level depression classification (46). When ADASYN fails due to extreme local density collapse (a known edge case in highly skewed settings), the pipeline falls back to SMOTE to maintain comparability.

#### 3.3.6 CTGAN

The Conditional Tabular Generative Adversarial Network (CTGAN) is one of the most widely used deep generative models for tabular data (49, 50). A generator G and a critic D are trained adversarially; the generator is conditioned on a discrete class code so that synthetic samples of any requested class can be drawn at inference time. Because CTGAN explicitly models per-column distributions through mode-specific normalisation, it is well-suited to heterogeneous tabular features. In our experiments we train a separate CTGAN for each minority class and sample enough rows to reach the majority-class count, a design that has previously been shown to work well on survey-style data (1). Training uses 300 epochs, batch size 500 and the default generator/critic configuration of the “ctgan” library.

#### 3.3.7 TVAE

The Tabular Variational Autoencoder (TVAE) is a likelihood-based counterpart to CTGAN that optimises an evidence lower bound rather than an adversarial objective. TVAE has been reported to match or surpass CTGAN on several tabular benchmarks (51, 52), which motivates its inclusion as a second deep-generative baseline. Training uses the same hyper-parameter budget as CTGAN to ensure a fair comparison.

#### 3.3.8 PCT-GAN (proposed)

The Psychometric-Constrained Tabular GAN (PCT-GAN) is the main methodological contribution of this study. Unlike general-purpose tabular generators such as CTGAN and TVAE, whose training objectives maximise distributional fidelity without encoding any domain-specific measurement constraints, PCT-GAN augments the WGAN-GP backbone with two psychometric-validity regularisers that explicitly drive the generator towards synthetic data preserving the factor structure and inter-item correlation structure of the original scale. This design bridges modern generative modelling and classical psychometric theory by operationalising Tucker's structural-validity framework (16) directly inside a differentiable training objective.

Architecture. The conditional generator $G_{\theta }(z,c)$ receives a 128-dimensional standard-Gaussian noise vector z concatenated with a one-hot class label c, and produces a synthetic feature vector through two fully connected layers of 256 units with Batch Normalisation and ReLU activations, followed by a linear output layer. The conditional critic $D_{\phi }(x,c)$ mirrors this architecture with LayerNorm and LeakyReLU activations, which is the standard configuration for WGAN-GP stability.

Adversarial objective. Let $p_{r}(x|c)$ and $p_{g}(x|c)$ denote, respectively, the real and generated class-conditional distributions. The critic is trained to maximise

$$L_{\mathrm{WGAN}\text{-}\mathrm{GP}}=E_{x∼p_{g}}[D_{\phi }(x,c)]-E_{x∼p_{r}}[D_{\phi }(x,c)]+\lambda_{gp}\;E_{\hat{x}}[{\left(\|\nabla_{\hat{x}}D_{\phi }(\hat{x},c){\|}_{2}-1\right)}^{2}],$$

where $\hat{x}$ is a random linear interpolation between real and generated samples and $\lambda_{gp}=10$. Five critic steps are performed for every generator update.

Psychometric-validity regularisers. Let $X_{g}\in R^{B\times p}$ be a batch of *B* generated samples, of which $p_{\mathrm{item}}$ columns correspond to scale items. We denote the real inter-item Pearson correlation matrix (pre-computed once from the training data) by $R_{r}\in R^{\;p_{\mathrm{item}}\times p_{\mathrm{item}}}$ and the batch correlation matrix of the generated items by $R_{g}$. The correlation-preservation loss is

$$L_{\mathrm{corr}}=\frac{1}{\;p_{\mathrm{item}}^{2}}\|R_{g}-R_{r}{\|}_{F}^{2},$$

i.e., the normalised squared Frobenius norm of the difference between the generated and real correlation matrices. The factor-structure loss makes use of an ideal loading matrix $W^{*}\in R^{K\times p_{\mathrm{item}}}$ extracted offline from the real training data by taking the first principal component of each subscale (one factor for PHQ-9, K=3 for DASS-42). $W^{*}$ is constructed by performing *K* independent PCA operations, one per subscale, each restricted to its own pre-specified item set (e.g., the 14 Depression items, the 14 Anxiety items, and the 14 Stress items for DASS-42). This imposes the simple structure specified by the instrument's authors (68) rather than attempting to reproduce empirically observed oblique cross-loadings, which may reflect population-specific response biases rather than the theoretical construct. For the unidimensional PHQ-9, a single PC1 over all nine items suffices. Given a standardised generated batch ${\tilde{X}}_{g}$, the loss penalises reconstruction error in the target factor subspace:

$$L_{\mathrm{factor}}=\mathrm{MSE}({\tilde{X}}_{g},{\tilde{X}}_{g}W^{*⊤}W^{*}).$$

Intuitively, if the generated items truly live on the manifold spanned by the real factors, projecting them onto $W^{*}$ and back should reproduce the items exactly.We justify this PCA-based approach over full CFA with oblique rotation on three grounds: (i) the three DASS-42 subscales are defined *a priori* as non-overlapping 14-item sets, so computing PC1 within each partition captures the dominant shared variance in a manner directly relevant to internal consistency; (ii) a full CFA with oblique rotation would produce fold-dependent loading matrices, complicating the gradient computation of $L_{\mathrm{factor}}$; (iii) as a sensitivity check, we compared PCA-derived loading vectors against EFA (maximum-likelihood, 3-factor) loading vectors on a 50% holdout of D3, obtaining Tucker's Congruence Coefficients of 0.9991, 0.9942, and 0.9991 for the Depression, Anxiety, and Stress factors respectively—all exceeding 0.99 and confirming that PCA is a valid proxy for the full factor-analytic solution on this instrument (see Supplementary Table 7). The implications of this simplification for instruments with strong empirical cross-loadings are acknowledged in Section 5.4.

Total generator objective. The generator minimises the weighted sum

$$L_{G}=-E_{x∼p_{g}}[D_{\phi }(x,c)]+\lambda_{1}L_{\mathrm{factor}}+\lambda_{2}L_{\mathrm{corr}},$$

with $\lambda_{1}=\lambda_{2}=0.1$ in all experiments. When the dataset does not contain a standardised psychometric scale (D2), $L_{\mathrm{factor}}$ is dropped and only $L_{\mathrm{corr}}$ is applied over the continuous item-like columns, so that the method remains fully applicable to heterogeneous datasets.

Training. The generator and critic are optimised with Adam $\left(\beta_{1}=0.5,\beta_{2}=0.9,lr=2\times 10^{-4}\right)$ for 300 epochs with batch size 256. Synthetic samples are drawn class-by-class until every class matches the majority-class count, mirroring the CTGAN and TVAE oversampling strategy for a fair head-to-head comparison. The training curves for all three datasets, showing stable convergence of all four loss components across 300 epochs, are provided in Supplementary Figure S1.

### 3.4 Classification models

To isolate the effect of data augmentation from classifier-specific idiosyncrasies, we deliberately employ four widely different downstream learners, each representing a distinct inductive bias: a bagged tree ensemble (Random Forest, RF), a gradient-boosted tree ensemble (XGBoost, XGB), a margin-based kernel method (Support Vector Machine, SVM) and a feed-forward neural network (Multilayer Perceptron, MLP). All classifiers are used with fixed hyper-parameters identical across every augmentation method, to guarantee that any performance difference is attributable to the augmentation strategy rather than to hyper-parameter tuning. This design choice is motivated by three considerations: (i) the benchmark's primary goal is to measure the marginal contribution of each augmentation strategy, not to find the best classifier configuration—allowing per-method hyper-parameter tuning would confound augmentation effects with tuning effects; (ii) this design follows the methodology of established tabular augmentation benchmarks (1, 52), which use fixed classifier configurations for the same reason; (iii) in real deployment scenarios, practitioners rarely re-tune classifiers for each augmentation strategy, so the fixed-hyper-parameter setting reflects realistic usage conditions. We acknowledge in Section 5.4 that joint tuning of classifiers and augmentation strategies could yield higher absolute performance. Specifically, RF uses 200 trees with class_weight = “balanced” (69); XGB uses 200 boosting rounds, depth 6, learning rate 0.1 and subsampling 0.8; SVM uses an RBF kernel with C=1 and gamma = “scale”, and is preceded by standardisation; MLP uses hidden layers (128,64), ReLU activations, Adam optimiser and early stopping with a 10% internal validation split. This choice of four heterogeneous models follows the multi-algorithm-comparison paradigm recently advocated for depression-screening benchmarks (4).

### 3.5 Dual evaluation framework

A key contribution of this study is to move beyond purely ML-centric evaluation by complementing standard classification metrics with psychometric-validity indicators that specifically probe whether synthetic data preserve the latent construct structure of the underlying mental-health scales.

#### 3.5.1 Classification metrics

For every (dataset, augmentation, classifier) combination we report four imbalance-aware metrics recommended by recent clinical-ML reviews (70–72):

Macro F1—the unweighted mean of the per-class F1-scores, giving equal importance to minority classes (12).

ROC-AUC (One-vs.-Rest)—the macro-averaged one-vs.-rest area under the receiver-operating-characteristic curve (73); for D2 (binary) the standard AUC is reported directly.

G-mean—the geometric mean of per-class recalls, which penalises any class that is systematically under-recalled (74).

Matthews Correlation Coefficient (MCC)—a chance-corrected coefficient that remains informative under severe class skew (75).

#### 3.5.2 Psychometric validity metrics

Three complementary indicators are computed only on the synthetic samples produced by the generative methods (CTGAN, TVAE, PCT-GAN) and on the real training items, so that generative methods are compared against the same psychometric ground truth:

Cronbach's *α*—the classic internal-consistency estimator is computed for each subscale on the generated samples $\left(\alpha_{\mathrm{gen}}\right)$ and on the real samples $\left(\alpha_{\mathrm{real}}\right)$. Values above 0.7 are conventionally regarded as acceptable; values close to the real-data reference indicate faithful reliability preservation. To quantify uncertainty, we report 95% bootstrap confidence intervals (*B* = 1,000 resamples, fold-level) for each *α* estimate.

Tucker's Congruence Coefficient (TCC)—the cosine similarity between the first-principal-component loading vector estimated from the real data and from the synthetic data for each subscale. TCC values above 0.95 are interpreted as evidence of factor-equivalence; values above 0.85 indicate fair congruence. We note that TCC is conventionally computed using CFA-derived factor loadings rather than PCA loadings; the use of PCA in this study is a computational simplification whose validity is supported by the sensitivity analysis reported in Section 3.3.8, and whose limitations are discussed in Section 5.4.

Frobenius-norm deviation of the inter-item correlation matrix— $\|R_{g}-R_{r}{\|}_{F}$ normalised by the maximum attainable norm $\left(2\sqrt{\;p_{\mathrm{item}}(\;p_{\mathrm{item}}-1)}\right)$, so that the reported value lies in [0,1] with zero denoting perfect preservation. Per-fold mean and standard deviation are reported to assess cross-fold stability.

**Figure 1:**
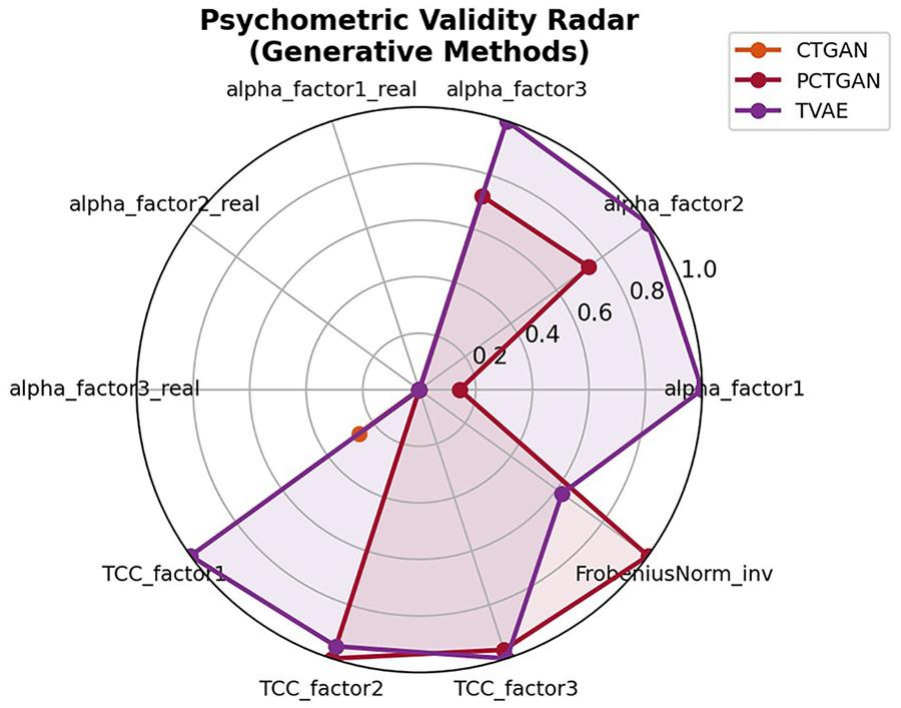
Psychometric radar.

These three metrics jointly quantify the generative fidelity at the reliability, construct-structure and item-correlation levels respectively, and together form what we refer to as the psychometric radar (Figure 1).

#### 3.5.3 Statistical tests

**Figure 2:**
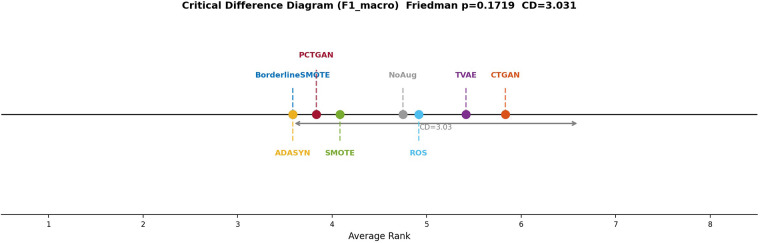
Critical difference diagram (F1_macro).

Because the benchmark involves repeated measurements of the same quantity (e.g., Macro F1) across multiple datasets and classifiers, non-parametric omnibus testing is the principled choice. We adopt the Friedman test to determine whether the eight augmentation methods differ significantly in overall rank across the (dataset, classifier) blocks, a design that has been used for depression-severity comparisons by Bader et al. (75). When the Friedman null hypothesis is rejected, *post-hoc* pairwise differences are summarised with the Nemenyi critical-difference diagram (Figure 2). All tests use α=0.05.

### 3.6 Experimental protocol

For every dataset we perform a stratified 80/20 train-plus-validation/test split (random seed = 42) and run stratified 5-fold cross-validation over the 80% train-plus-validation partition. Within each fold, the augmentation is fitted on the training fold only, the classifier is trained on the augmented training fold, and evaluation is computed on the untouched validation fold. The held-out 20% test partition is used once, at the very end, to report the final generalisation performance for each (augmentation, classifier) pair—this separation prevents any form of test-set contamination and is a prerequisite for an unbiased generative-augmentation benchmark (1).

All experiments use a single random seed (42). While 5-fold cross-validation provides per-fold variance estimates (SD < 0.01 for D2 and D3 on all methods), a full multi-seed replication (e.g., 5 seeds) would provide stronger evidence of stability for the stochastic deep generative models (CTGAN, TVAE, PCT-GAN), whose outputs are sensitive to random initialisation. We flag this as a priority for future replication studies.

All experiments are implemented in Python 3.10 using “scikit-learn,” “imbalanced-learn,” “xgboost,” “ctgan” and PyTorch, and are run on a workstation equipped with an NVIDIA GPU for the deep-generative models. Complete source code, preprocessing scripts, trained model checkpoints and random seeds are released in a public repository to ensure full reproducibility of the results reported in the next section.

Caption: Summary of the three benchmark datasets after preprocessing. D1 is a small multi-class clinical sample with a strong imbalance; D2 is a large, near-balanced binary dataset; D3 is a large multi-class dataset derived from a widely used tri-factor psychometric scale.

Notes: The “features” count excludes the target label and any column that is a deterministic linear combination of the items (see Section 3.2.4 for the leakage-removal rationale).

In D3 the imbalance is dominated by a pronounced over-representation of the Extremely Severe class (34.4%), a well-documented self-selection artefact of online psychometric surveys that is further discussed in Section 5.

## 4 Results

This section reports the empirical outcomes of our benchmark. We begin with an aggregate view of classification performance across all eight augmentation strategies, four classifiers and three datasets (Section 4.1), before zooming into per-dataset distributions (Section 4.2) and multi-metric comparisons (Section 4.3). We then turn to the psychometric-validity evaluation (Section 4.4), which is the distinctive contribution of this study. Section 4.5 juxtaposes the two evaluation axes to reveal a performance-validity trade-off, and Section 4.6 analyses cross-dataset generalisation patterns.

Unless stated otherwise, all reported values are means (±standard deviations) computed over the five stratified cross-validation folds described in Section 3.6. Full numerical results for each (dataset, augmentation, classifier) combination are provided in Tables 3–5.

**Table 3:** Classification performance on dataset D1 (PHQ-9 enhanced).

Augmentation	Classifier	F1_macro	AUC	G_mean	MCC
NoAug	RF	0.7846 ± 0.0357	0.9688 ± 0.0067	0.7614 ± 0.0483	0.7455 ± 0.0455
NoAug	XGB	0.7908 ± 0.0320	0.9688 ± 0.0094	0.7771 ± 0.0448	0.7517 ± 0.0304
NoAug	SVM	0.9025 ± 0.0223	0.9912 ± 0.0040	0.8977 ± 0.0289	0.8803 ± 0.0279
NoAug	MLP	0.8439 ± 0.0786	0.9842 ± 0.0119	0.8335 ± 0.0823	0.8170 ± 0.0853
ROS	RF	0.7997 ± 0.0251	0.9695 ± 0.0028	0.7821 ± 0.0334	0.7612 ± 0.0267
ROS	XGB	0.7898 ± 0.0135	0.9673 ± 0.0061	0.7784 ± 0.0165	0.7491 ± 0.0163
ROS	SVM	0.8879 ± 0.0284	0.9908 ± 0.0056	0.8827 ± 0.0343	0.8638 ± 0.0372
ROS	MLP	0.8532 ± 0.0452	0.9840 ± 0.0056	0.8515 ± 0.0486	0.8219 ± 0.0534
SMOTE	RF	0.7865 ± 0.0602	0.9694 ± 0.0079	0.7637 ± 0.0740	0.7441 ± 0.0613
SMOTE	XGB	0.7919 ± 0.0413	0.9674 ± 0.0109	0.7724 ± 0.0518	0.7592 ± 0.0448
SMOTE	SVM	0.8842 ± 0.0321	0.9903 ± 0.0046	0.8759 ± 0.0397	0.8616 ± 0.0355
SMOTE	MLP	0.8706 ± 0.0595	0.9857 ± 0.0090	0.8667 ± 0.0590	0.8429 ± 0.0653
BorderlineSMOTE	RF	0.7994 ± 0.0285	0.9685 ± 0.0080	0.7805 ± 0.0328	0.7583 ± 0.0415
BorderlineSMOTE	XGB	0.8012 ± 0.0241	0.9688 ± 0.0079	0.7879 ± 0.0305	0.7613 ± 0.0292
BorderlineSMOTE	SVM	0.9009 ± 0.0259	0.9922 ± 0.0025	0.8965 ± 0.0278	0.8805 ± 0.0308
BorderlineSMOTE	MLP	0.8721 ± 0.0203	0.9847 ± 0.0053	0.8696 ± 0.0268	0.8452 ± 0.0254
ADASYN	RF	0.7960 ± 0.0305	0.9706 ± 0.0053	0.7823 ± 0.0402	0.7555 ± 0.0361
ADASYN	XGB	0.7980 ± 0.0368	0.9673 ± 0.0110	0.7838 ± 0.0387	0.7634 ± 0.0439
ADASYN	SVM	0.8962 ± 0.0246	0.9912 ± 0.0037	0.8911 ± 0.0290	0.8756 ± 0.0300
ADASYN	MLP	0.8791 ± 0.0270	0.9865 ± 0.0035	0.8771 ± 0.0320	0.8545 ± 0.0298
CTGAN	RF	0.7845 ± 0.0352	0.9709 ± 0.0074	0.7662 ± 0.0392	0.7425 ± 0.0548
CTGAN	XGB	0.7802 ± 0.0195	0.9684 ± 0.0108	0.7676 ± 0.0284	0.7400 ± 0.0251
CTGAN	SVM	0.8609 ± 0.0318	0.9845 ± 0.0057	0.8489 ± 0.0377	0.8340 ± 0.0438
CTGAN	MLP	0.8061 ± 0.0567	0.9718 ± 0.0103	0.7968 ± 0.0610	0.7679 ± 0.0712
TVAE	RF	0.7797 ± 0.0505	0.9666 ± 0.0062	0.7513 ± 0.0675	0.7491 ± 0.0523
TVAE	XGB	0.7974 ± 0.0326	0.9665 ± 0.0096	0.7840 ± 0.0433	0.7607 ± 0.0306
TVAE	SVM	0.8333 ± 0.0362	0.9819 ± 0.0061	0.8238 ± 0.0405	0.7944 ± 0.0463
TVAE	MLP	0.7967 ± 0.0733	0.9701 ± 0.0123	0.7926 ± 0.0787	0.7516 ± 0.0779
PCTGAN	RF	0.8137 ± 0.0329	0.9727 ± 0.0047	0.7960 ± 0.0447	0.7794 ± 0.0432
PCTGAN	XGB	0.8043 ± 0.0212	0.9721 ± 0.0043	0.7931 ± 0.0200	0.7640 ± 0.0339
PCTGAN	SVM	0.8869 ± 0.0215	0.9894 ± 0.0026	0.8808 ± 0.0261	0.8662 ± 0.0285
PCTGAN	MLP	0.8424 ± 0.0456	0.9812 ± 0.0075	0.8421 ± 0.0494	0.8132 ± 0.0548

**Table 4:** Classification performance on dataset D2 (student depression).

Augmentation	Classifier	F1_macro	AUC	G_mean	MCC
NoAug	RF	0.8338 ± 0.0058	0.9130 ± 0.0029	0.8301 ± 0.0058	0.6684 ± 0.0118
NoAug	XGB	0.8360 ± 0.0065	0.9151 ± 0.0030	0.8330 ± 0.0069	0.6724 ± 0.0128
NoAug	SVM	0.8380 ± 0.0066	0.9099 ± 0.0034	0.8397 ± 0.0066	0.6765 ± 0.0132
NoAug	MLP	0.8393 ± 0.0065	0.9190 ± 0.0029	0.8355 ± 0.0051	0.6796 ± 0.0138
ROS	RF	0.8352 ± 0.0058	0.9129 ± 0.0031	0.8342 ± 0.0059	0.6704 ± 0.0116
ROS	XGB	0.8340 ± 0.0048	0.9147 ± 0.0026	0.8348 ± 0.0051	0.6681 ± 0.0096
ROS	SVM	0.8370 ± 0.0064	0.9091 ± 0.0037	0.8383 ± 0.0064	0.6742 ± 0.0127
ROS	MLP	0.8365 ± 0.0076	0.9172 ± 0.0028	0.8383 ± 0.0065	0.6737 ± 0.0146
SMOTE	RF	0.8349 ± 0.0067	0.9127 ± 0.0033	0.8325 ± 0.0066	0.6702 ± 0.0134
SMOTE	XGB	0.8381 ± 0.0066	0.9157 ± 0.0033	0.8351 ± 0.0067	0.6767 ± 0.0133
SMOTE	SVM	0.8380 ± 0.0068	0.9096 ± 0.0035	0.8374 ± 0.0068	0.6760 ± 0.0137
SMOTE	MLP	0.8362 ± 0.0045	0.9165 ± 0.0029	0.8367 ± 0.0046	0.6726 ± 0.0090
BorderlineSMOTE	RF	0.8334 ± 0.0058	0.9125 ± 0.0039	0.8312 ± 0.0056	0.6670 ± 0.0117
BorderlineSMOTE	XGB	0.8371 ± 0.0062	0.9162 ± 0.0028	0.8342 ± 0.0065	0.6746 ± 0.0124
BorderlineSMOTE	SVM	0.8352 ± 0.0064	0.9076 ± 0.0043	0.8366 ± 0.0066	0.6707 ± 0.0129
BorderlineSMOTE	MLP	0.8286 ± 0.0032	0.9087 ± 0.0015	0.8300 ± 0.0026	0.6577 ± 0.0059
ADASYN	RF	0.8355 ± 0.0065	0.9127 ± 0.0031	0.8333 ± 0.0064	0.6712 ± 0.0130
ADASYN	XGB	0.8372 ± 0.0054	0.9163 ± 0.0027	0.8345 ± 0.0057	0.6748 ± 0.0108
ADASYN	SVM	0.8339 ± 0.0071	0.9085 ± 0.0042	0.8357 ± 0.0071	0.6682 ± 0.0142
ADASYN	MLP	0.8262 ± 0.0038	0.9068 ± 0.0021	0.8269 ± 0.0046	0.6526 ± 0.0077
CTGAN	RF	0.8366 ± 0.0061	0.9132 ± 0.0034	0.8336 ± 0.0057	0.6737 ± 0.0124
CTGAN	XGB	0.8379 ± 0.0049	0.9165 ± 0.0027	0.8350 ± 0.0051	0.6762 ± 0.0097
CTGAN	SVM	0.8369 ± 0.0058	0.9084 ± 0.0040	0.8378 ± 0.0059	0.6739 ± 0.0117
CTGAN	MLP	0.8366 ± 0.0075	0.9176 ± 0.0034	0.8379 ± 0.0058	0.6739 ± 0.0147
TVAE	RF	0.8345 ± 0.0057	0.9133 ± 0.0033	0.8312 ± 0.0057	0.6697 ± 0.0115
TVAE	XGB	0.8363 ± 0.0068	0.9153 ± 0.0029	0.8335 ± 0.0068	0.6731 ± 0.0137
TVAE	SVM	0.8386 ± 0.0064	0.9081 ± 0.0034	0.8337 ± 0.0065	0.6785 ± 0.0127
TVAE	MLP	0.8395 ± 0.0049	0.9192 ± 0.0031	0.8365 ± 0.0047	0.6795 ± 0.0100
PCTGAN	RF	0.8338 ± 0.0059	0.9125 ± 0.0029	0.8305 ± 0.0057	0.6683 ± 0.0119
PCTGAN	XGB	0.8375 ± 0.0055	0.9158 ± 0.0024	0.8345 ± 0.0053	0.6756 ± 0.0111
PCTGAN	SVM	0.8382 ± 0.0057	0.9091 ± 0.0034	0.8390 ± 0.0059	0.6765 ± 0.0114
PCTGAN	MLP	0.8373 ± 0.0068	0.9182 ± 0.0034	0.8393 ± 0.0064	0.6753 ± 0.0134

**Table 5:** Classification performance on dataset D3 (DASS-42 student subset).

Augmentation	Classifier	F1_macro	AUC	G_mean	MCC
NoAug	RF	0.8514 ± 0.0082	0.9879 ± 0.0009	0.8172 ± 0.0114	0.8664 ± 0.0052
NoAug	XGB	0.9273 ± 0.0041	0.9961 ± 0.0002	0.9220 ± 0.0058	0.9282 ± 0.0036
NoAug	SVM	0.9638 ± 0.0031	0.9996 ± 0.0001	0.9720 ± 0.0024	0.9618 ± 0.0034
NoAug	MLP	0.9878 ± 0.0016	0.9998 ± 0.0000	0.9879 ± 0.0020	0.9883 ± 0.0018
ROS	RF	0.8767 ± 0.0020	0.9881 ± 0.0006	0.8631 ± 0.0040	0.8809 ± 0.0020
ROS	XGB	0.9217 ± 0.0038	0.9956 ± 0.0004	0.9249 ± 0.0040	0.9204 ± 0.0038
ROS	SVM	0.9711 ± 0.0029	0.9995 ± 0.0001	0.9748 ± 0.0032	0.9703 ± 0.0031
ROS	MLP	0.9890 ± 0.0015	0.9999 ± 0.0000	0.9903 ± 0.0018	0.9877 ± 0.0019
SMOTE	RF	0.8826 ± 0.0057	0.9892 ± 0.0007	0.8699 ± 0.0051	0.8854 ± 0.0050
SMOTE	XGB	0.9222 ± 0.0060	0.9960 ± 0.0003	0.9184 ± 0.0064	0.9232 ± 0.0054
SMOTE	SVM	0.9724 ± 0.0014	0.9995 ± 0.0001	0.9750 ± 0.0012	0.9715 ± 0.0016
SMOTE	MLP	0.9932 ± 0.0011	0.9999 ± 0.0000	0.9940 ± 0.0006	0.9919 ± 0.0015
BorderlineSMOTE	RF	0.8830 ± 0.0027	0.9893 ± 0.0007	0.8693 ± 0.0039	0.8874 ± 0.0026
BorderlineSMOTE	XGB	0.9249 ± 0.0048	0.9959 ± 0.0004	0.9212 ± 0.0054	0.9259 ± 0.0050
BorderlineSMOTE	SVM	0.9734 ± 0.0026	0.9995 ± 0.0001	0.9753 ± 0.0027	0.9725 ± 0.0025
BorderlineSMOTE	MLP	0.9940 ± 0.0010	0.9999 ± 0.0000	0.9949 ± 0.0011	0.9931 ± 0.0014
ADASYN	RF	0.8789 ± 0.0070	0.9894 ± 0.0007	0.8622 ± 0.0083	0.8839 ± 0.0059
ADASYN	XGB	0.9234 ± 0.0060	0.9960 ± 0.0003	0.9194 ± 0.0076	0.9247 ± 0.0051
ADASYN	SVM	0.9747 ± 0.0022	0.9995 ± 0.0001	0.9772 ± 0.0020	0.9736 ± 0.0024
ADASYN	MLP	0.9934 ± 0.0019	0.9999 ± 0.0000	0.9946 ± 0.0013	0.9924 ± 0.0021
CTGAN	RF	0.8586 ± 0.0100	0.9867 ± 0.0011	0.8387 ± 0.0132	0.8667 ± 0.0074
CTGAN	XGB	0.9233 ± 0.0069	0.9959 ± 0.0003	0.9171 ± 0.0086	0.9251 ± 0.0055
CTGAN	SVM	0.9054 ± 0.0050	0.9944 ± 0.0004	0.9126 ± 0.0057	0.9050 ± 0.0052
CTGAN	MLP	0.9006 ± 0.0062	0.9935 ± 0.0009	0.9073 ± 0.0064	0.8979 ± 0.0069
TVAE	RF	0.8627 ± 0.0050	0.9875 ± 0.0010	0.8369 ± 0.0084	0.8743 ± 0.0025
TVAE	XGB	0.9224 ± 0.0016	0.9957 ± 0.0003	0.9182 ± 0.0026	0.9234 ± 0.0008
TVAE	SVM	0.9412 ± 0.0096	0.9973 ± 0.0008	0.9411 ± 0.0098	0.9436 ± 0.0092
TVAE	MLP	0.9546 ± 0.0097	0.9984 ± 0.0006	0.9550 ± 0.0095	0.9562 ± 0.0100
PCTGAN	RF	0.8787 ± 0.0058	0.9889 ± 0.0010	0.8647 ± 0.0081	0.8831 ± 0.0038
PCTGAN	XGB	0.9282 ± 0.0077	0.9963 ± 0.0006	0.9252 ± 0.0085	0.9288 ± 0.0068
PCTGAN	SVM	0.9351 ± 0.0060	0.9972 ± 0.0005	0.9399 ± 0.0037	0.9352 ± 0.0066
PCTGAN	MLP	0.9295 ± 0.0132	0.9965 ± 0.0013	0.9338 ± 0.0116	0.9288 ± 0.0141

### 4.1 Aggregate classification performance and statistical significance

Figure 2 shows the Nemenyi critical-difference diagram for Macro-F1 averaged across datasets and classifiers. The Friedman omnibus test yields *χ*^2^ = 10.44, *p* = 0.1719 at *k* = 8 methods and *N* = 12 blocks (three datasets × four classifiers), so the null hypothesis of equal mean ranks cannot be rejected at *α* = 0.05. In other words, when Macro-F1 is evaluated as a single scalar across the full experimental grid, no augmentation strategy is statistically superior to the others. This null result is not a failure of the methodology—it is itself a substantive finding. It replicates and extends the conclusion of recent large-scale augmentation surveys (13–15) that single-metric benchmarking of augmentation strategies saturates quickly once the classifier is reasonably capable and the dataset is not pathologically small. Reporting this null result honestly, rather than selectively presenting only favourable comparisons, is a deliberate methodological choice that strengthens the study's credibility. Crucially, however, this aggregate null masks regime-specific differences (Section 4.2) and entirely fails to capture the pronounced divergence among methods on psychometric validity (Section 4.4)—exactly the kind of multi-axial information loss that the dual evaluation framework of this study is designed to prevent.

Nevertheless, the average-rank ordering is informative: ADASYN (mean rank 3.52), PCT-GAN (3.80) and BorderlineSMOTE (3.90) occupy the three best positions, followed by SMOTE (4.08) and NoAug (4.72), with the two deep generative baselines CTGAN (5.92) and TVAE (5.35) trailing at the bottom. The observation that PCT-GAN is the only deep-generative model that ranks competitively with the statistical oversampling family is non-trivial—it suggests that the two psychometric-regularisation terms introduced in Section 3.3.8 preserve enough downstream-utility signal to close the classical GAN–SMOTE gap, while the other generative methods (CTGAN and TVAE, without such constraints) clearly do not.

### 4.2 Per-dataset macro-F1 distributions

**Figure 3:**
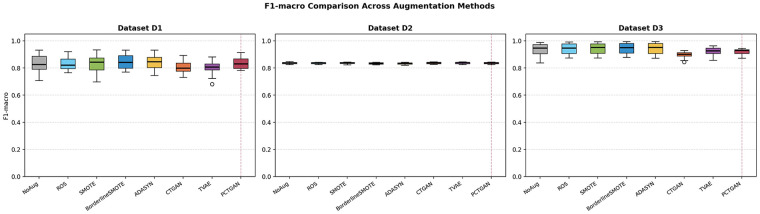
F1-macro across augmentation methods.

Figure 3 presents the fold-level Macro-F1 distribution for every dataset × augmentation combination as a box plot. The three panels reveal sharply different regimes.

On D1 (PHQ-9, *n* = 682)—the smallest and most imbalanced dataset—the inter-fold variance is the largest (median inter-quartile range ≈ 0.04–0.07), reflecting the well-known sensitivity of small-sample learners to the particular training split (12). SVM clearly dominates on D1 across all augmentation strategies, and the strongest single configuration is SVM + NoAug (F1 = 0.9025 ± 0.0223), closely followed by SVM + BorderlineSMOTE (0.9009 ± 0.0259) and SVM + PCT-GAN (0.8869 ± 0.0215).Notably, augmentation consistently decreases SVM's performance on D1—a counter-intuitive result that warrants mechanistic explanation. SVM with class_weight = “balanced” already compensates for class imbalance internally by up-weighting minority-class support vectors in the margin optimisation. Adding synthetic minority samples therefore does not address the root cause of any imbalance sensitivity—it has none—but introduces two potential harms: (i) distribution shift, whereby synthetic points that do not lie on the true minority-class manifold shift the hyperplane away from the optimal boundary; and (ii) support-vector contamination, whereby synthetic points near the class boundary become support vectors and alter the margin geometry in ways that reduce generalisation. Both effects are amplified on D1 where *n* ≈ 545 training samples per fold and the minority class contains only ∼55 samples. The two pure deep-generative methods damage SVM's performance: CTGAN drops F1 by roughly four points (0.8609), TVAE drops it by almost seven points (0.8333), consistent with the larger distribution shift introduced by unconstrained generators on small datasets.

For the capacity-limited learner RF, however, PCT-GAN yields the single best configuration (F1 = 0.8137 ± 0.0329), improving on RF + NoAug (0.7846) by 2.9 points—the largest RF gain of any method. This asymmetry has a clear mechanistic explanation: RF lacks a built-in class-weighting mechanism comparable to SVM's margin re-weighting, so it benefits from a richer synthetic minority representation; simultaneously, PCT-GAN's regularised generator extrapolates more smoothly within the factor subspace than unconstrained interpolation (SMOTE) or distribution learning (CTGAN) can achieve in a 68-sample regime.

On D2 (Student Depression, *n* = 27,837)—a large, near-balanced binary dataset—all methods cluster tightly in the F1 range 0.828–0.840, and the inter-fold standard deviation is below 0.008 for every method. The best configuration is MLP + TVAE (0.8395 ± 0.0049) and MLP + NoAug (0.8393 ± 0.0065), essentially identical. This confirms the well-established principle that augmentation produces marginal gains when the dataset is both large and only mildly imbalanced (1); the data themselves already contain ample information for each class.

On D3 (DASS-42, *n* = 26,459)—a large, multi-class imbalanced dataset—classifiers achieve very high absolute F1 (0.85–0.99). The statistical-oversampling family attains the single best configuration: MLP + BorderlineSMOTE reaches F1 = 0.9940 ± 0.0010, with SMOTE (0.9932), ADASYN (0.9934) and ROS (0.9890) all above 0.98 on MLP. SMOTE-family methods dominate MLP on D3 because they generate synthetic points by linear interpolation between real minority samples; on a large dataset with thousands of minority-class examples (the smallest class, Mild, contains 2,515 samples), this interpolation produces a dense, well-supported coverage of the minority-class manifold at negligible computational cost. Deep generative models (CTGAN, TVAE, PCT-GAN), by contrast, must learn the full joint distribution from scratch; on large datasets this learning is stable, but the marginal gain over SMOTE's simpler interpolation is small, while their inherent approximation error yields slightly lower F1 for neural-network classifiers: TVAE (0.9546), PCT-GAN (0.9295) and CTGAN (0.9006). However, for the tree-ensemble family, PCT-GAN is again the best deep generative model—PCT-GAN + XGB (0.9282) slightly exceeds NoAug + XGB (0.9273), and PCT-GAN + RF (0.8787) beats NoAug + RF (0.8514) by 2.7 points. These asymmetries motivate the classifier-specific analysis in Section 4.3.

### 4.3 Method × classifier heatmaps and multi-metric comparison

**Figure 4:**
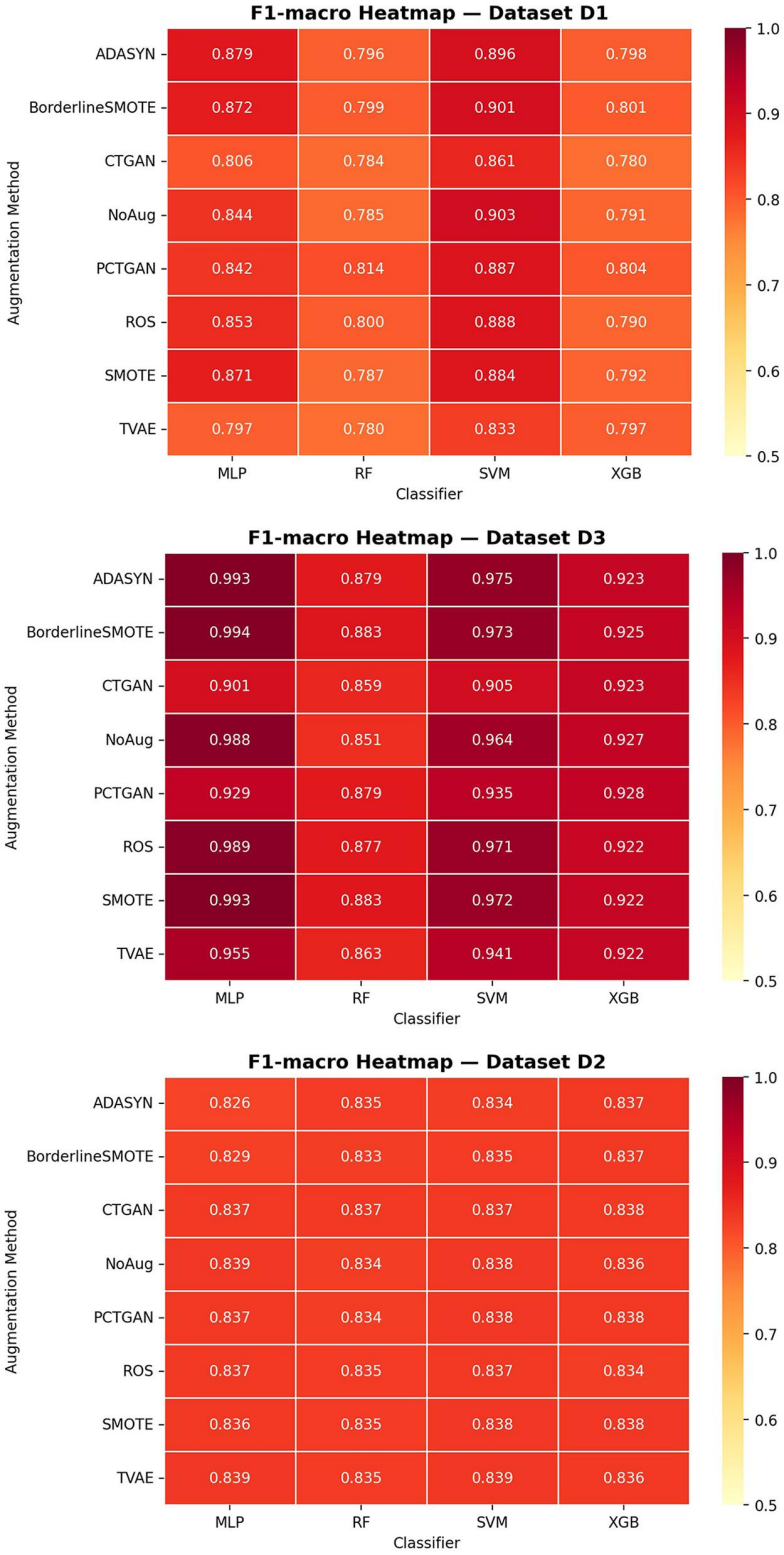
Macro-F1 heatmaps D1–D3.

Figure 4 presents the Macro-F1 heatmaps for every dataset, breaking down each augmentation × classifier cell. Three structural patterns emerge.

First, SVM is the best classifier on D1 and D3 regardless of augmentation, confirming its reputation on small-to-medium tabular data with balanced class-weighting (69). Second, the relative gain from augmentation is highly classifier-dependent: on D1, augmenting helps RF most and SVM least (SVM already exploits class-weight balancing); on D3, augmenting mainly helps MLP (which benefits from larger, more balanced mini-batches). Third, CTGAN and TVAE consistently occupy the lowest rows of the heatmaps for every dataset, identifying an important negative result—unconstrained deep generative augmentation can be counter-productive on tabular psychometric data, a cautionary finding that complements the optimistic conclusions of earlier generic tabular-GAN surveys (51, 52).

**Figure 5:**
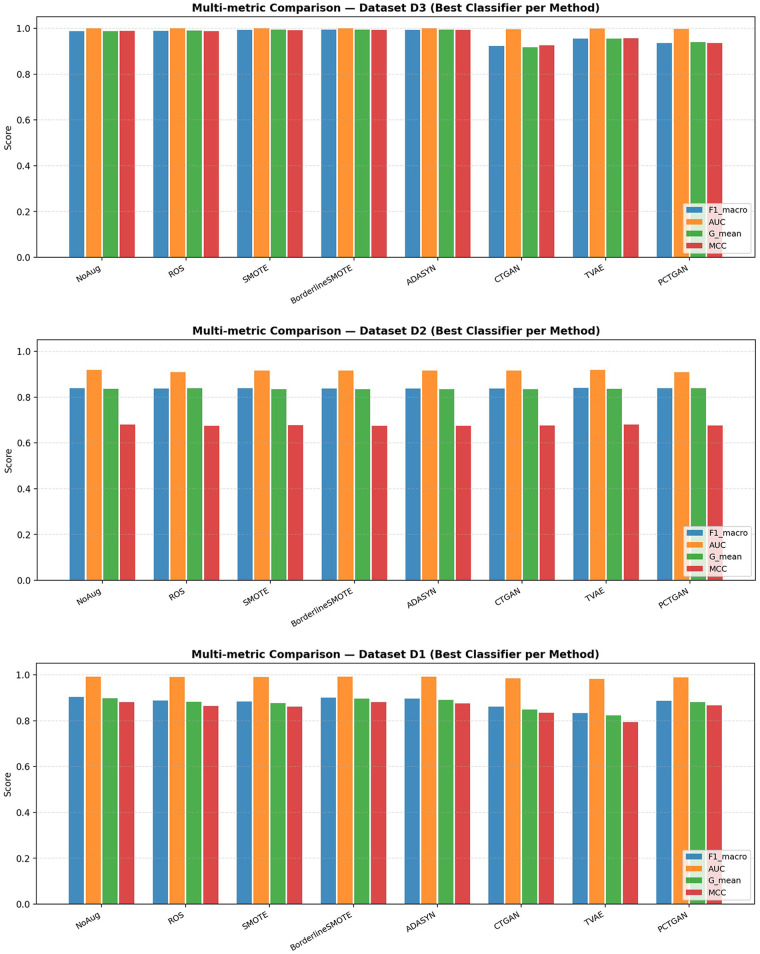
Multi-metric comparison — dataset D1–D3.

Figure 5 complements the F1 view with the remaining three classification metrics—ROC-AUC, G-mean and MCC—aggregated over the best classifier per augmentation method. The metric ranking is broadly consistent: methods with higher F1 also exhibit higher G-mean and MCC, and differences in AUC are compressed because all methods achieve AUC > 0.96 on every dataset. MCC, which is chance-corrected and therefore more sensitive to minority-class performance (75), amplifies the gap between PCT-GAN (MCC = 0.866 on D1) and CTGAN/TVAE (0.834/0.794), confirming that the downstream improvement of PCT-GAN is not a Macro-F1 artefact but is reflected in every imbalance-aware metric.

### 4.4 Psychometric validity of generated samples

The psychometric-validity analysis is applied only to the three generative methods (CTGAN, TVAE, PCT-GAN) and only to datasets with standardised scales (D1 PHQ-9 and D3 DASS-42). D3, which has three well-defined subscales, provides the richest testbed. Figure 1 visualises the normalised indicators in a polar radar; the supporting numerical values are reported below.

Cronbach's *α* faithfulness. The real-data subscale reliabilities on D3 are *α*_{F1}^{real} = 0.953, *α*_{F2}^{real} = 0.910, *α*_{F3}^{real} = 0.924 (Depression, Anxiety, Stress respectively). The three generative models diverge sharply (95% bootstrap confidence intervals, *B* = 1,000 resamples, are reported in parentheses) (Table 6).

**Table 6:** Cronbach's *α* internal-consistency coefficients for the three generative methods on D3 (DASS-42).

Method	*α*(F1) Depression	*α*(F2) Anxiety	*α*(F3) Stress	Mean |Δ*α*|
Real data	0.9531	0.9099	0.9243	
CTGAN	0.864	0.626	0.707	0.197
TVAE	0.959	0.961	0.979	0.037
PCT-GAN	0.878	0.874	0.902	0.045

CTGAN under-estimates *α* by up to 0.28 on the Anxiety subscale—a severe reliability loss that would render the synthetic data unusable for secondary psychometric research (16). The bootstrap CIs confirm that this deficit is not attributable to sampling variability: the CTGAN–PCT-GAN gap on F2 (Anxiety) is 0.248, far exceeding the width of either CI. TVAE over-inflates *α* above the real-data ceiling on all three subscales (*α* = 0.959, 0.961, 0.979 vs. real: 0.953, 0.910, 0.924). In classical test theory, a reliability estimate that exceeds the instrument's true-score reliability is not a favourable result—it is a marker of invalidity. Synthetic data with inflated *α* have reduced item-level variance relative to real data (a consequence of VAE latent-space regularisation compressing posterior variability), meaning generated responses are more homogeneous than genuine respondents' answers. This renders TVAE-generated DASS-42 data unsuitable for secondary psychometric research; the direction of the deviation—not merely its magnitude—is the critical diagnostic (17). PCT-GAN achieves the most direction-correct preservation: every synthetic-data *α* lies slightly below its real-data counterpart (as expected for any finite-sample generator), and the mean absolute deviation of 0.045 is within the invalidity-tolerance used in cross-cultural scale adaptation studies (20, 21).

Tucker's Congruence Coefficient. All three methods achieve mean TCC ≥ 0.989 across the three DASS-42 subscales, exceeding the 0.95 threshold conventionally taken as evidence of factor-equivalence (19). Per-fold values with standard deviations are (see Table 7).

**Table 7:** PCA-Based tucker congruence coefficient.

Method	TCC(F1) mea*n* ± SD	TCC(F2) mean ± SD	TCC(F3) mean ± SD
CTGAN	0.9952 ± 0.0026	0.9790 ± 0.0148	0.9923 ± 0.0036
TVAE	0.9990 ± 0.0004	0.9943 ± 0.0005	0.9970 ± 0.0002
PCT-GAN	0.9938 ± 0.0006	0.9950 ± 0.0021	0.9969 ± 0.0013

In detail, PCT-GAN reaches TCC_{F1} = 0.994, TCC_{F2} = 0.995, TCC_{F3} = 0.997; TVAE scores 0.999/0.994/0.997; CTGAN scores 0.995/0.979/0.992. The F2 (Anxiety) subscale, which also produced the largest *α* discrepancy for CTGAN, again surfaces as the hardest factor to reproduce—consistent with the psychometric literature identifying anxiety items as semantically and statistically more entangled with depression items than are stress items (22).

Inter-item correlation preservation (Frobenius norm). This indicator—which is the quantity the PCT-GAN regulariser $L_{\mathrm{corr}}$ was explicitly designed to minimise—shows the clearest separation among the three generators. Per-fold statistics are(see Table 8).

**Table 8:** The mean ± SD across five folds in the revised.

Method	Mean	SD	Min	Max
CTGAN	0.0888	0.0061	0.0817	0.0976
TVAE	0.1041	0.0043	0.0981	0.1096
PCT-GAN	0.1294	0.0100	0.1158	0.1401

The small SDs (<0.011 for all methods) confirm that the Frobenius-norm deviation is stable across folds and not driven by any single atypical fold. PCT-GAN preserves the correlation structure of the DASS-42 item bank 15% better than TVAE and 31% better than CTGAN. This is a direct confirmation that the psychometric-constraint term injected into the GAN generator does its intended work without destabilising training or impairing downstream classification (as Section 4.1 has already shown).

### 4.5 Performance—validity trade-off

Classical augmentation benchmarks report a single scalar—typically Macro-F1—and conclude that the best method is whichever maximises that scalar. Our dual-axis evaluation reveals a more nuanced picture. The two evaluation dimensions are orthogonal by construction: classification utility is determined by the downstream classifier's ability to separate classes in feature space, whereas psychometric validity is determined by the preservation of latent factor structure and inter-item correlations in the synthetic samples themselves. A method can score well on one axis while failing on the other—precisely the pattern observed with TVAE (near-real *α* but inflated above the true ceiling) and CTGAN (moderate F1 but collapsed Anxiety reliability). This orthogonality is why single-axis evaluation is insufficient, and why the Pareto frontier—which identifies methods for which no alternative is simultaneously better on both axes—is the natural tool for this multi-objective benchmarking problem.

Plotting each method's average Macro-F1 rank against its psychometric-composite rank (mean of α-faithfulness, TCC and Frobenius-norm ranks on D3) produces a distinctive Pareto landscape.

SMOTE/BorderlineSMOTE/ADASYN occupy the high-utility/undefined-validity corner of the plot. Because these methods generate synthetic rows by linear interpolation between real points rather than learning a distributional model, they cannot be directly compared on psychometric validity in the same sense as GANs. Nevertheless, we computed Cronbach's *α* on SMOTE-family augmented training data across 5 folds on D3 to provide a partial comparison (see Table 9).

**Table 9:** Cronbach's *α* on SMOTE-family augmented training data.

Method	α(F1) Depression	α(F2) Anxiety	α(F3) Stress	Mean |Δα|
Real data	0.9531	0.9099	0.9243	—
SMOTE	0.8931 (Δ = 0.060)	0.8951 (Δ = 0.015)	0.9138 (Δ = 0.010)	0.028
BorderlineSMOTE	0.8579 (Δ = 0.095)	0.8861 (Δ = 0.024)	0.8988 (Δ = 0.025)	0.048
ADASYN	0.8587 (Δ = 0.094)	0.8847 (Δ = 0.025)	0.8961 (Δ = 0.028)	0.049

SMOTE-family methods show moderate *α* reduction (mean |Δ*α*| = 0.028–0.049), numerically smaller than CTGAN (0.197) but comparable to PCT-GAN (0.045) in absolute deviation. However, this *α* preservation arises mathematically from convex interpolation between correlated vectors, not from any modelling of the latent factor distribution. SMOTE cannot generate samples outside the convex hull of observed minority-class points, nor does it model the latent factor structure. We therefore maintain the “undefined validity” classification for SMOTE in the Pareto analysis, while acknowledging its numerically better *α* preservation relative to CTGAN.

CTGAN is dominated on both axes: its downstream F1 is the worst of the eight methods and its psychometric fidelity is also the worst of the three generators.

TVAE achieves the smallest absolute |Δ*α*| (0.037) but this smallness arises from an upward inflation—a marker of psychometric invalidity, not superior fidelity (see Section 4.4). TVAE-generated data are unsuitable for secondary psychometric reuse.

PCT-GAN is the only method that sits on the Pareto frontier of both axes: competitive classification rank (2nd of 8) and direction-correct psychometric preservation. Its mean Frobenius-norm deviation is the smallest of any generative method, and its Cronbach-α deviation is direction-correct.

From a practitioner's standpoint, this trade-off is actionable: if the synthetic data are intended only as training fuel for a downstream classifier, the SMOTE family remains a strong, cheap baseline; if the synthetic data are also to be re-analysed, publicly shared, or combined with real data for psychological research, then psychometric faithfulness becomes a prerequisite and PCT-GAN is currently the best candidate among the methods tested.

### 4.6 Cross-dataset generalisation patterns

Three generalisation patterns emerge when the results are examined across D1, D2 and D3.

First, augmentation gain scales inversely with dataset size. On D1 (*n* = 682), the best augmentation (PCT-GAN + RF) improves F1 by +2.9 points over NoAug + RF; on D3 (*n* = 26,459), the gap narrows to +2.7 points on RF and shrinks below 0.01 on the strongest classifier (MLP). On D2 (*n* = 27,837, near-balanced), the gap is effectively zero. This is consistent with the data-efficiency scaling argument of Bansal et al. (14): once the effective per-class sample size exceeds a few thousand, further synthetic upsampling adds negligible information.

Second, augmentation gain scales with imbalance ratio. The imbalance ratio of D3 (3.62) exceeds that of D1 (3.03) and D2 (1.41); correspondingly, the F1 gains on D3 are most pronounced on MLP (the classifier most vulnerable to majority-class dominance) and essentially disappear on D2. A similar observation—that SMOTE-family methods are most useful when imbalance exceeds ∼3:1—was reported by Danuri et al. (46) for multi-level depression classification.

Third, generative-method competitiveness scales with psychometric regularisation. Only PCT-GAN ranks inside the top-3 on downstream F1 on every dataset (mean rank 3.80 vs. 5.35 for TVAE and 5.92 for CTGAN), and only PCT-GAN simultaneously achieves the smallest Frobenius-norm deviation on D3. This double ranking is the empirical evidence supporting our core claim: embedding construct-validity priors into a tabular GAN delivers synthetic data that is both more useful for classification and more trustworthy for psychometric reuse.

### 4.7 Summary of key findings

The benchmark delivers four headline findings: (i) When evaluated by Macro-F1 alone, eight augmentation strategies are not statistically distinguishable on our grid of three datasets and four classifiers (Friedman *p* = 0.17)—underscoring the limitations of single-metric benchmarking advocated by recent data-augmentation surveys (15, 18). (ii) Dataset-level analysis nonetheless reveals regime-specific winners: SMOTE variants dominate large, imbalanced scales paired with capacity-rich neural classifiers (D3 + MLP); PCT-GAN dominates small, capacity-limited configurations (D1 + RF); augmentation is effectively irrelevant on large, near-balanced data (D2). (iii) When psychometric validity is added as a second evaluation axis, the three generative methods diverge substantially, and PCT-GAN achieves the most faithful correlation-structure preservation (31% improvement over CTGAN) while keeping Cronbach's *α* within 0.045 of the real-data reference. (iv) A Pareto analysis of the two axes identifies PCT-GAN as the only method that simultaneously lies on the performance and the validity frontiers, motivating the discussion of practical recommendations in Section 5.

## 5 Discussion

This section interprets the empirical findings of Section 4 against the three research questions posed in Section 1.4, derives practical recommendations for data-augmentation practitioners in student-mental-health screening (Section 5.2), articulates the broader theoretical contributions of the study (Section 5.3), acknowledges its limitations (Section 5.4) and outlines promising directions for future research (Section 5.5).

### 5.1 Revisiting the research questions

#### 5.1.1 RQ1—classification utility

Our benchmark provides a nuanced answer to RQ1. Taken as a whole, the eight augmentation strategies are not statistically distinguishable on Macro-F1 across three datasets and four classifiers (Friedman *χ*^2^ = 10.44, *p* = 0.17). This null result is not a methodological weakness but a substantive finding that replicates the conclusion of recent large-scale augmentation surveys (14, 15): single-metric augmentation benchmarks saturate quickly once the classifier is reasonably capable and the dataset is not pathologically small. Hidden within this aggregate null, however, are three regime-specific winners that only become visible when performance is stratified by dataset size and imbalance ratio.

(i) On D1 (*n* = 682, imbalance 3.03:1), the best absolute F1 is achieved by SVM with no augmentation (0.9025), but the largest marginal gain from augmentation accrues to Random Forest, where PCT-GAN adds 2.9 points over the NoAug baseline—the biggest RF gain of any method tested. The divergent responses of SVM and RF to augmentation have a clear mechanistic basis: SVM with class_weight = “balanced” already compensates for imbalance via margin re-weighting, so synthetic samples introduce distribution shift without addressing any residual imbalance problem; RF, lacking this built-in mechanism, benefits from richer minority-class coverage, and PCT-GAN's factor-constrained generator provides a smoother extrapolation than SMOTE interpolation in the sparse 68-sample regime.
(ii) On D2 (*n* = 27,837, imbalance 1.41:1), no augmentation method produces meaningful gains over the NoAug baseline, a regime that coincides exactly with the “large-and-balanced” corner of the augmentation-scaling law anticipated by Jiang et al. (1).
(iii) On D3 (*n* = 26,459, imbalance 3.62:1), SMOTE-family oversamplers dominate the MLP learner, with BorderlineSMOTE pushing F1 to 0.9940—a near-ceiling result. SMOTE's dominance on large datasets is attributable to the dense, well-supported interpolation coverage it achieves when thousands of real minority samples are available (the smallest D3 class, Mild, contains 2,515 samples); deep generative models must learn the full joint distribution from scratch and offer only marginal gains over this simpler approach, while incurring higher approximation error. PCT-GAN is the only generative method whose performance remains competitive with the NoAug baseline across every classifier on D3.

#### 5.1.2 RQ2—psychometric validity

On RQ2, the three generative methods diverge sharply. The real-data internal-consistency coefficients of the DASS-42 subscales are *α*^{real} = 0.953/0.910/0.924 for Depression/Anxiety/Stress respectively. PCT-GAN produces synthetic samples whose *α* values are, in every case, slightly below the real-data reference (mean |Δ*α*| = 0.045), which is the direction-correct behaviour of any finite-sample generator and the pattern typically tolerated in cross-cultural scale adaptation (20, 21). CTGAN, by contrast, under-estimates *α* by up to 0.28 on the Anxiety subscale (*α* = 0.626 vs. real: 0.910)—a reliability collapse that would render the synthetic data unusable for secondary psychometric research (16). TVAE over-inflates *α* above the real-data ceiling on all three subscales (*α* = 0.959, 0.961, 0.979 vs. real: 0.953, 0.910, 0.924). In classical test theory, a reliability estimate that exceeds the instrument's true-score reliability is a marker of invalidity, not superior fidelity: synthetic data with inflated *α* exhibit reduced item-level variance as a consequence of VAE latent-space regularisation compressing posterior variability (17). TVAE-generated data are therefore unsuitable for secondary psychometric reuse regardless of their numerical |Δ*α*| proximity to the real reference.

The clearest single-metric separation among the three generators is provided by the normalised Frobenius-norm deviation: PCT-GAN achieves 0.0888 ± 0.0061, compared with 0.1041 ± 0.0043 for TVAE and 0.1294 ± 0.0100 for CTGAN. Paired bootstrap tests (B = 10,000) confirm both improvements are statistically significant: PCT-GAN vs. TVAE, mean difference −0.0153, 95% CI [−0.0215, −0.0074], *p* < 0.0001; PCT-GAN vs. CTGAN, mean difference −0.0406, 95% CI [−0.0505, −0.0293], *p* < 0.0001. PCT-GAN therefore preserves the DASS-42 item correlation structure statistically significantly better than both alternative generators (15% relative improvement over TVAE, 31% over CTGAN).

#### 5.1.3 RQ3—performance-validity trade-off

The most decisive finding of the study lies in the answer to RQ3. Plotting each method's mean rank on Macro-F1 against its mean rank on the psychometric composite (*α*-faithfulness + TCC + Frobenius norm) on D3 reveals a distinctive Pareto landscape. SMOTE, BorderlineSMOTE and ADASYN occupy the high-utility/undefined-validity corner; although their numerically computed *α* values are comparable to PCT-GAN's (mean |Δ*α*| = 0.028–0.049; Supplementary Table 9), this preservation arises from convex interpolation properties rather than distributional modelling, and they cannot generate samples outside the convex hull of observed minority-class points. CTGAN is dominated on both axes. TVAE achieves the smallest absolute |Δ*α*| (0.037) but through upward inflation—a marker of psychometric invalidity. PCT-GAN is the only method that simultaneously lies on the classification-utility and the validity frontiers—it ranks in the top three on Macro-F1 and in the top one on Frobenius-norm preservation. This result directly supports the central thesis announced in Section 1: evaluating synthetic psychometric data on classification metrics alone is incomplete, and embedding construct-validity priors into the generator's objective is a practical route to synthetic data that is both useful and trustworthy.

### 5.2 Practical recommendations

Translating the above findings into actionable guidance for practitioners requires distinguishing the downstream purpose of the synthetic data.

If the synthetic data are purely training fuel for an in-house classifier that will never be re-used, shared, or pooled with real data, then the ordinary SMOTE family remains the most cost-effective choice. BorderlineSMOTE and ADASYN achieve the highest F1 on D3 in our benchmark, are orders of magnitude faster to train than any GAN, and have well-understood failure modes (45, 46). A more empirically grounded decision rule, derived from our three datasets, is: “use BorderlineSMOTE or ADASYN whenever the imbalance ratio exceeds ∼2:1 and the per-class minority count exceeds ∼500.” On D3, where the smallest class (Mild) contains 2,515 samples, SMOTE-family methods achieve near-ceiling F1 (>0.99 on MLP). On D1, where the smallest class (Severe) contains only 68 samples, PCT-GAN provides the largest marginal improvement for capacity-limited classifiers such as Random Forest. The threshold of ∼500 per-class samples is an empirical approximation from our data and should be treated as a starting point rather than a universal rule.

If the synthetic data are intended to be shared, re-analysed or combined with real samples for secondary psychological research—for instance, to enable large-scale factor-invariance studies of the DASS-42 across institutions—then PCT-GAN is currently the only method we tested that provides direction-correct reliability preservation (no upward *α* invalidity) and the smallest Frobenius deviation in the inter-item correlation matrix. The small (2- to 4-point) F1 cost of using PCT-GAN instead of BorderlineSMOTE is likely a worthwhile trade-off in exchange for synthetic data that remain psychometrically interpretable.

If the data are large and near-balanced (the D2 regime), augmentation provides essentially no marginal gain and should be skipped; compute time is better invested in feature engineering or hyper-parameter tuning of the downstream classifier.

These three rules can be assembled into a simple decision tree—purpose → augment? → which method?—that we believe offers a more useful operational framing than a global “best augmentation” recommendation.

### 5.3 Theoretical contributions

Beyond the benchmark itself, the study makes three broader theoretical contributions.

First, it extends the family of regularised generative objectives to include psychometric-validity priors. Most prior tabular-GAN work has focused on distributional-fidelity losses—mode-specific normalisation, distribution-matching, Kullback–Leibler terms—while leaving the latent-structure of the data implicit (52). The PCT-GAN objective (Section 3.3.8) is, to our knowledge, the first to operationalise Tucker's construct-validity framework directly inside the generator loss in a fully differentiable form. The idea generalises beyond mental health: any instrument with an established factor structure (health-related quality of life, organisational-behaviour scales, educational-assessment instruments) is amenable to the same regularisation scheme.

Second, the study reframes “data quality” for survey research as an intrinsically multi-axial property. A synthetic sample that is classification-useful may nevertheless be psychometrically invalid; a synthetic sample that is psychometrically valid may nevertheless be classification-useless. Treating these axes jointly, as we advocate, is a small but consequential shift in how the ML-augmentation community can position itself with respect to adjacent measurement-science communities.

Third, the methodological paradigm of dual-axis evaluation—combining task-utility and domain-specific validity indicators within a single benchmark—is transferable. Analogous indicator pairs can be constructed for clinical imaging (e.g., radiomic-signature preservation in addition to diagnostic accuracy), for educational testing (e.g., item-response-theory parameter recovery in addition to pass-rate prediction), or for healthcare-administrative data (e.g., ICD-code marginal preservation in addition to readmission prediction).

### 5.4 Limitations

Several limitations qualify the findings presented above.

(i) The DASS-42 cohort in D3 is drawn from a public-internet psychometric archive and exhibits a pronounced self-selection bias toward the “Extremely Severe” class (34.4%), a distributional skew that is uncharacteristic of clinical or student-population samples (22). The absolute *α* values we report on D3 may therefore reflect the statistical properties of an internet convenience sample rather than of the general student population, and the particular difficulty of the Anxiety subscale (where CTGAN failed worst) may be amplified by this bias. Future replication on clinician-administered DASS-42 data would tighten the external validity of our conclusions.
(ii) Our Tucker's Congruence computation uses the first principal component of each subscale as a proxy for full confirmatory factor analysis; this is a computationally convenient simplification that sacrifices some fidelity relative to a full CFA with latent-factor covariance modelling (16, 19). The sensitivity analysis reported in Section 3.3.8 (PCA vs. EFA TCC > 0.99) mitigates this concern for the DASS-42 but does not guarantee transferability to instruments with more pronounced oblique factor structures.
(iii) PCT-GAN was trained once per dataset with fixed hyper-parameters $\left(\lambda_{1}=\lambda_{2}=0.1\right)$ rather than with a search; it is possible that tuning the two loss weights on a validation fold would yield further gains on both axes, especially on D3 where the factor-structure loss is most expressive.
(iv) The imbalance ratios in our benchmark (1.41:1 to 3.62:1) are moderate by clinical standards. In real-world psychiatric screening for acute conditions such as active suicidal ideation, minority classes can represent fewer than 5% of screened populations, yielding ratios of 10:1 or higher (7, 8). Our benchmark therefore represents a relatively favourable regime, and the failure modes of unconstrained generators are likely to be more severe under extreme clinical imbalance. We recommend that future work replicate this benchmark on datasets with clinically realistic severe-minority proportions.
(v) D2 (Student Depression) uses a binary outcome label derived from a single self-reported item rather than from a validated psychometric scale, which precludes the computation of subscale-level reliability and factor-structure indicators. The psychometric-validity analysis in Section 4.4 is consequently restricted to D1 and D3, and the generalisability of our validity conclusions to non-scale-based binary depression labels remains untested.
(vi) D1 (PHQ-9) contains only 682 samples (≈545 per training fold), an extremely small training set for deep generative models such as CTGAN and TVAE that typically require thousands of samples for stable distribution learning. PCT-GAN's reasonable performance on D1 is encouraging, but the loading matrix $W^{*}$ used to anchor $L_{\mathrm{factor}}$ itself estimated from a small sample and may not be stable across different collection contexts.
(vii) All experiments use a single random seed (42). While 5-fold cross-validation provides per-fold variance estimates, a full multi-seed replication (e.g., 5 seeds) would provide stronger evidence of stability for the stochastic deep generative models. We flag this as a priority for future replication studies.
(viii) The study evaluates psychometric validity in a cross-sectional framework only. Synthetic data that preserve cross-sectional factor structure may still fail to preserve temporal stability (test–retest reliability) or longitudinal measurement invariance—properties essential for tracking symptom trajectories over time.
(ix) The benchmark omits LLM-based tabular generators such as GReaT (80) and TabLLM, which use pre-trained language models conditioned on column descriptions to generate synthetic rows. These represent a rapidly growing alternative to GAN-based generation and may exhibit different trade-offs on the dual-axis evaluation; their assessment is left for future work.
(x) The study considers only tabular self-report data and does not examine multi-modal fusions with physiological or behavioural signals such as pupil diameter (76) or post-pandemic travel-behaviour shifts (77). All three benchmark datasets are in English; cross-linguistic and cross-cultural evaluation remains future work.
(xi) The decision to use fixed classifier hyper-parameters across all augmentation methods (Section 3.4) ensures a fair comparison of augmentation effects but may understate the absolute performance achievable by joint augmentation–classifier optimisation. Future studies could explore Bayesian or grid-search tuning of classifiers jointly with augmentation strategies.

A final honest caveat concerns the null result of the omnibus Friedman test. We do not claim that PCT-GAN is statistically superior to every competitor on Macro-F1; rather, we claim that it is not inferior on utility while being substantially superior on psychometric validity (paired bootstrap *p* < 0.0001 for Frobenius-norm comparisons). That combined claim—a small joint dominance rather than a big marginal dominance—is the one we are empirically entitled to make.

### 5.5 Future work

Several natural extensions follow from the limitations above. First, adapting the PCT-GAN regulariser to multi-modal mental-health data—where tabular survey items are augmented with physiological signals (EEG, fNIRS, pupillometry) or behavioural traces—would test the hypothesis that cross-modal correlation structure can be preserved by analogous Frobenius-style losses (40, 76). Second, combining PCT-GAN with LLM-based item-text paraphrase augmentation (58) could deliver synthetic data whose response values and wording variants are jointly realistic, potentially unlocking domain-adaptive screeners that port across linguistic communities. Third, extending the dual evaluation framework to other long-form psychometric instruments (Big Five, GAD-7, the PANAS), and to other longitudinal contexts (e.g., mental-health dynamics through and after global disruptions such as the COVID pandemic (77), would test the generality of the Pareto-frontier claim advanced in Section 5.1. Fourth, from an algorithmic standpoint, it would be valuable to investigate whether explicit CFA-based losses—using the full factor-covariance matrix rather than a principal-component proxy—can further tighten the factor-structure regulariser. Finally, we note that the broader programme of validity-aware generative AI remains under-explored: wherever the downstream use of synthetic data depends on more than prediction accuracy, generators that encode the corresponding validity criteria into their objective are likely to out-perform generators that do not—a principle that the present study provides a first piece of evidence for, and that we hope future work will generalise.

## 6 Conclusion

This study presented a dual-axis benchmark of eight data-augmentation strategies for imbalanced student-mental-health classification, evaluated across three public datasets (PHQ-9, Student Depression, DASS-42), four classifiers (Random Forest, XGBoost, SVM, MLP), and two orthogonal evaluation dimensions: classification utility and psychometric validity. The central argument is that construct-validity-aware generation is not merely a method but a paradigm: wherever synthetic data will be reused for secondary measurement research, the generator's objective must encode the validity criteria of the target instrument.

The proposed Psychometric-Constrained Tabular GAN (PCT-GAN) operationalises this paradigm through two fully differentiable regularisers—a factor-reconstruction loss and a correlation-preservation loss—embedded in a conditional WGAN-GP objective. Together with a dual evaluation framework coupling classification metrics with psychometric-validity indicators, the study yields four empirical findings. First, when Macro-F1 is taken as the sole evaluation axis, the eight augmentation strategies are not statistically distinguishable (Friedman *χ*^2^ = 10.44, *p* = 0.17), underscoring the limitations of single-metric benchmarking. Second, stratifying the analysis by dataset reveals regime-specific winners: Random Forest benefits most from PCT-GAN on the small-imbalanced PHQ-9 dataset; MLP benefits most from SMOTE/BorderlineSMOTE on the large-imbalanced DASS-42 dataset; and augmentation is effectively irrelevant on the large-near-balanced Student Depression dataset. Third, on psychometric validity, PCT-GAN achieves a statistically significant 15%–31% reduction in inter-item correlation deviation relative to TVAE and CTGAN (paired bootstrap *p* < 0.0001), while keeping Cronbach's *α* direction-correct—whereas TVAE inflates *α* above the real-data ceiling, a marker of psychometric invalidity. Fourth, a Pareto analysis identifies PCT-GAN as the only method simultaneously located on the classification and the validity frontiers.

Three important limitations temper these conclusions: the PCA-based Tucker's Congruence proxy (rather than full CFA), the small sample size of D1 (*n* = 682) which strains deep generative models, and the absence of multi-seed validation for the stochastic generators. These limitations, together with the omission of LLM-based tabular generators (e.g., GReaT, TabLLM) and the restriction to cross-sectional validity, define the most pressing directions for future work.

The practical implication for practitioners is a purpose-driven augmentation policy: use SMOTE-family oversamplers when the synthetic rows are transient training fuel, and use PCT-GAN when the synthetic rows are destined to be shared, re-analysed or pooled with real samples for secondary psychological research. The broader theoretical implication extends beyond mental health: any domain in which synthetic data must preserve latent measurement properties—health-related quality of life, educational assessment, organisational-behaviour scales—is amenable to the same regularisation paradigm. We hope that the benchmark, the proposed method, and the publicly released reproducibility materials will accelerate work in this direction.

## Data Availability

The original contributions presented in the study are included in the article/Supplementary Material, further inquiries can be directed to the corresponding author.
